# Search for production of four top quarks in final states with same-sign or multiple leptons in proton–proton collisions at $$\sqrt{s}=13$$$$\,\text {TeV}$$

**DOI:** 10.1140/epjc/s10052-019-7593-7

**Published:** 2020-01-31

**Authors:** A. M. Sirunyan, A. Tumasyan, W. Adam, F. Ambrogi, T. Bergauer, J. Brandstetter, M. Dragicevic, J. Erö, A. Escalante Del Valle, M. Flechl, R. Frühwirth, M. Jeitler, N. Krammer, I. Krätschmer, D. Liko, T. Madlener, I. Mikulec, N. Rad, J. Schieck, R. Schöfbeck, M. Spanring, D. Spitzbart, W. Waltenberger, C.-E. Wulz, M. Zarucki, V. Drugakov, V. Mossolov, J. Suarez Gonzalez, M. R. Darwish, E. A. De Wolf, D. Di Croce, X. Janssen, A. Lelek, M. Pieters, H. Rejeb Sfar, H. Van Haevermaet, P. Van Mechelen, S. Van Putte, N. Van Remortel, F. Blekman, E. S. Bols, S. S. Chhibra, J. D’Hondt, J. De Clercq, D. Lontkovskyi, S. Lowette, I. Marchesini, S. Moortgat, Q. Python, K. Skovpen, S. Tavernier, W. Van Doninck, P. Van Mulders, D. Beghin, B. Bilin, H. Brun, B. Clerbaux, G. De Lentdecker, H. Delannoy, B. Dorney, L. Favart, A. Grebenyuk, A. K. Kalsi, A. Popov, N. Postiau, E. Starling, L. Thomas, C. Vander Velde, P. Vanlaer, D. Vannerom, T. Cornelis, D. Dobur, I. Khvastunov, M. Niedziela, C. Roskas, D. Trocino, M. Tytgat, W. Verbeke, B. Vermassen, M. Vit, N. Zaganidis, O. Bondu, G. Bruno, C. Caputo, P. David, C. Delaere, M. Delcourt, A. Giammanco, V. Lemaitre, A. Magitteri, J. Prisciandaro, A. Saggio, M. Vidal Marono, P. Vischia, J. Zobec, F. L. Alves, G. A. Alves, G. Correia Silva, C. Hensel, A. Moraes, P. Rebello Teles, E. Belchior Batista Das Chagas, W. Carvalho, J. Chinellato, E. Coelho, E. M. Da Costa, G. G. Da Silveira, D. De Jesus Damiao, C. De Oliveira Martins, S. Fonseca De Souza, L. M. Huertas Guativa, H. Malbouisson, J. Martins, D. Matos Figueiredo, M. Medina Jaime, M. Melo De Almeida, C. Mora Herrera, L. Mundim, H. Nogima, W. L. Prado Da Silva, L. J. Sanchez Rosas, A. Santoro, A. Sznajder, M. Thiel, E. J. Tonelli Manganote, F. Torres Da Silva De Araujo, A. Vilela Pereira, C. A. Bernardes, L. Calligaris, T. R. Fernandez Perez Tomei, E. M. Gregores, D. S. Lemos, P. G. Mercadante, S. F. Novaes, SandraS. Padula, A. Aleksandrov, G. Antchev, R. Hadjiiska, P. Iaydjiev, M. Misheva, M. Rodozov, M. Shopova, G. Sultanov, M. Bonchev, A. Dimitrov, T. Ivanov, L. Litov, B. Pavlov, P. Petkov, W. Fang, X. Gao, L. Yuan, M. Ahmad, G. M. Chen, H. S. Chen, M. Chen, C. H. Jiang, D. Leggat, H. Liao, Z. Liu, A. Spiezia, J. Tao, E. Yazgan, H. Zhang, S. Zhang, J. Zhao, A. Agapitos, Y. Ban, G. Chen, A. Levin, J. Li, L. Li, Q. Li, Y. Mao, S. J. Qian, D. Wang, Q. Wang, Z. Hu, Y. Wang, M. Xiao, C. Avila, A. Cabrera, C. Florez, C. F. González Hernández, M. A. Segura Delgado, J. Mejia Guisao, J. D. Ruiz Alvarez, C. A. Salazar González, N. Vanegas Arbelaez, D. Giljanović, N. Godinovic, D. Lelas, I. Puljak, T. Sculac, Z. Antunovic, M. Kovac, V. Brigljevic, S. Ceci, D. Ferencek, K. Kadija, B. Mesic, M. Roguljic, A. Starodumov, T. Susa, M. W. Ather, A. Attikis, E. Erodotou, A. Ioannou, M. Kolosova, S. Konstantinou, G. Mavromanolakis, J. Mousa, C. Nicolaou, F. Ptochos, P. A. Razis, H. Rykaczewski, D. Tsiakkouri, M. Finger, M. Finger, A. Kveton, J. Tomsa, E. Ayala, E. Carrera Jarrin, S. Elgammal, E. Salama, S. Bhowmik, A. Carvalho Antunes De Oliveira, R. K. Dewanjee, K. Ehataht, M. Kadastik, M. Raidal, C. Veelken, P. Eerola, L. Forthomme, H. Kirschenmann, K. Osterberg, M. Voutilainen, F. Garcia, J. Havukainen, J. K. Heikkilä, T. Järvinen, V. Karimäki, M. S. Kim, R. Kinnunen, T. Lampén, K. Lassila-Perini, S. Laurila, S. Lehti, T. Lindén, P. Luukka, T. Mäenpää, H. Siikonen, E. Tuominen, J. Tuominiemi, T. Tuuva, M. Besancon, F. Couderc, M. Dejardin, D. Denegri, B. Fabbro, J. L. Faure, F. Ferri, S. Ganjour, A. Givernaud, P. Gras, G. Hamel de Monchenault, P. Jarry, C. Leloup, E. Locci, J. Malcles, J. Rander, A. Rosowsky, M. Ö. Sahin, A. Savoy-Navarro, M. Titov, S. Ahuja, C. Amendola, F. Beaudette, P. Busson, C. Charlot, B. Diab, G. Falmagne, R. Granier de Cassagnac, I. Kucher, A. Lobanov, C. Martin Perez, M. Nguyen, C. Ochando, P. Paganini, J. Rembser, R. Salerno, J. B. Sauvan, Y. Sirois, A. Zabi, A. Zghiche, J.-L. Agram, J. Andrea, D. Bloch, G. Bourgatte, J.-M. Brom, E. C. Chabert, C. Collard, E. Conte, J.-C. Fontaine, D. Gelé, U. Goerlach, M. Jansová, A.-C. Le Bihan, N. Tonon, P. Van Hove, S. Gadrat, S. Beauceron, C. Bernet, G. Boudoul, C. Camen, A. Carle, N. Chanon, R. Chierici, D. Contardo, P. Depasse, H. El Mamouni, J. Fay, S. Gascon, M. Gouzevitch, B. Ille, Sa. Jain, F. Lagarde, I. B. Laktineh, H. Lattaud, A. Lesauvage, M. Lethuillier, L. Mirabito, S. Perries, V. Sordini, L. Torterotot, G. Touquet, M. Vander Donckt, S. Viret, A. Khvedelidze, Z. Tsamalaidze, C. Autermann, L. Feld, M. K. Kiesel, K. Klein, M. Lipinski, D. Meuser, A. Pauls, M. Preuten, M. P. Rauch, J. Schulz, M. Teroerde, B. Wittmer, A. Albert, M. Erdmann, B. Fischer, S. Ghosh, T. Hebbeker, K. Hoepfner, H. Keller, L. Mastrolorenzo, M. Merschmeyer, A. Meyer, P. Millet, G. Mocellin, S. Mondal, S. Mukherjee, D. Noll, A. Novak, T. Pook, A. Pozdnyakov, T. Quast, M. Radziej, Y. Rath, H. Reithler, J. Roemer, A. Schmidt, S. C. Schuler, A. Sharma, S. Wiedenbeck, S. Zaleski, G. Flügge, W. Haj Ahmad, O. Hlushchenko, T. Kress, T. Müller, A. Nehrkorn, A. Nowack, C. Pistone, O. Pooth, D. Roy, H. Sert, A. Stahl, M. Aldaya Martin, P. Asmuss, I. Babounikau, H. Bakhshiansohi, K. Beernaert, O. Behnke, A. Bermúdez Martínez, D. Bertsche, A. A. Bin Anuar, K. Borras, V. Botta, A. Campbell, A. Cardini, P. Connor, S. Consuegra Rodríguez, C. Contreras-Campana, V. Danilov, A. De Wit, M. M. Defranchis, C. Diez Pardos, D. Domínguez Damiani, G. Eckerlin, D. Eckstein, T. Eichhorn, A. Elwood, E. Eren, E. Gallo, A. Geiser, A. Grohsjean, M. Guthoff, M. Haranko, A. Harb, A. Jafari, N. Z. Jomhari, H. Jung, A. Kasem, M. Kasemann, H. Kaveh, J. Keaveney, C. Kleinwort, J. Knolle, D. Krücker, W. Lange, T. Lenz, J. Leonard, J. Lidrych, K. Lipka, W. Lohmann, R. Mankel, I.-A. Melzer-Pellmann, A. B. Meyer, M. Meyer, M. Missiroli, G. Mittag, J. Mnich, A. Mussgiller, V. Myronenko, D. Pérez Adán, S. K. Pflitsch, D. Pitzl, A. Raspereza, A. Saibel, M. Savitskyi, V. Scheurer, P. Schütze, C. Schwanenberger, R. Shevchenko, A. Singh, H. Tholen, O. Turkot, A. Vagnerini, M. Van De Klundert, R. Walsh, Y. Wen, K. Wichmann, C. Wissing, O. Zenaiev, R. Zlebcik, R. Aggleton, S. Bein, L. Benato, A. Benecke, V. Blobel, T. Dreyer, A. Ebrahimi, F. Feindt, A. Fröhlich, C. Garbers, E. Garutti, D. Gonzalez, P. Gunnellini, J. Haller, A. Hinzmann, A. Karavdina, G. Kasieczka, R. Klanner, R. Kogler, N. Kovalchuk, S. Kurz, V. Kutzner, J. Lange, T. Lange, A. Malara, J. Multhaup, C. E. N. Niemeyer, A. Perieanu, A. Reimers, O. Rieger, C. Scharf, P. Schleper, S. Schumann, J. Schwandt, J. Sonneveld, H. Stadie, G. Steinbrück, F. M. Stober, B. Vormwald, I. Zoi, M. Akbiyik, C. Barth, M. Baselga, S. Baur, T. Berger, E. Butz, R. Caspart, T. Chwalek, W. De Boer, A. Dierlamm, K. El Morabit, N. Faltermann, M. Giffels, P. Goldenzweig, A. Gottmann, M. A. Harrendorf, F. Hartmann, U. Husemann, I. Katkov, S. Kudella, S. Mitra, M. U. Mozer, D. Müller, Th. Müller, M. Musich, A. Nürnberg, G. Quast, K. Rabbertz, M. Schröder, I. Shvetsov, H. J. Simonis, R. Ulrich, M. Wassmer, M. Weber, C. Wöhrmann, R. Wolf, G. Anagnostou, P. Asenov, G. Daskalakis, T. Geralis, A. Kyriakis, D. Loukas, G. Paspalaki, M. Diamantopoulou, G. Karathanasis, P. Kontaxakis, A. Manousakis-katsikakis, A. Panagiotou, I. Papavergou, N. Saoulidou, A. Stakia, K. Theofilatos, K. Vellidis, E. Vourliotis, G. Bakas, K. Kousouris, I. Papakrivopoulos, G. Tsipolitis, I. Evangelou, C. Foudas, P. Gianneios, P. Katsoulis, P. Kokkas, S. Mallios, K. Manitara, N. Manthos, I. Papadopoulos, J. Strologas, F. A. Triantis, D. Tsitsonis, M. Bartók, R. Chudasama, M. Csanad, P. Major, K. Mandal, A. Mehta, M. I. Nagy, G. Pasztor, O. Surányi, G. I. Veres, G. Bencze, C. Hajdu, D. Horvath, F. Sikler, T. Vámi, V. Veszpremi, G. Vesztergombi, N. Beni, S. Czellar, J. Karancsi, A. Makovec, J. Molnar, Z. Szillasi, P. Raics, D. Teyssier, Z. L. Trocsanyi, B. Ujvari, T. Csorgo, W. J. Metzger, F. Nemes, T. Novak, S. Choudhury, J. R. Komaragiri, P. C. Tiwari, S. Bahinipati, C. Kar, G. Kole, P. Mal, V. K. Muraleedharan Nair Bindhu, A. Nayak, D. K. Sahoo, S. K. Swain, S. Bansal, S. B. Beri, V. Bhatnagar, S. Chauhan, R. Chawla, N. Dhingra, R. Gupta, A. Kaur, M. Kaur, S. Kaur, P. Kumari, M. Lohan, M. Meena, K. Sandeep, S. Sharma, J. B. Singh, A. K. Virdi, G. Walia, A. Bhardwaj, B. C. Choudhary, R. B. Garg, M. Gola, S. Keshri, Ashok Kumar, S. Malhotra, M. Naimuddin, P. Priyanka, K. Ranjan, Aashaq Shah, R. Sharma, R. Bhardwaj, M. Bharti, R. Bhattacharya, S. Bhattacharya, U. Bhawandeep, D. Bhowmik, S. Dutta, S. Ghosh, M. Maity, K. Mondal, S. Nandan, A. Purohit, P. K. Rout, G. Saha, S. Sarkar, T. Sarkar, M. Sharan, B. Singh, S. Thakur, P. K. Behera, P. Kalbhor, A. Muhammad, P. R. Pujahari, A. Sharma, A. K. Sikdar, D. Dutta, V. Jha, V. Kumar, D. K. Mishra, P. K. Netrakanti, L. M. Pant, P. Shukla, T. Aziz, M. A. Bhat, S. Dugad, G. B. Mohanty, N. Sur, RavindraKumar Verma, S. Banerjee, S. Bhattacharya, S. Chatterjee, P. Das, M. Guchait, S. Karmakar, S. Kumar, G. Majumder, K. Mazumdar, N. Sahoo, S. Sawant, S. Chauhan, S. Dube, V. Hegde, B. Kansal, A. Kapoor, K. Kothekar, S. Pandey, A. Rane, A. Rastogi, S. Sharma, S. Chenarani, E. Eskandari Tadavani, S. M. Etesami, M. Khakzad, M. Mohammadi Najafabadi, M. Naseri, F. Rezaei Hosseinabadi, M. Felcini, M. Grunewald, M. Abbrescia, R. Aly, C. Calabria, A. Colaleo, D. Creanza, L. Cristella, N. De Filippis, M. De Palma, A. Di Florio, L. Fiore, A. Gelmi, G. Iaselli, M. Ince, S. Lezki, G. Maggi, M. Maggi, G. Miniello, S. My, S. Nuzzo, A. Pompili, G. Pugliese, R. Radogna, A. Ranieri, G. Selvaggi, L. Silvestris, F. M. Simone, R. Venditti, P. Verwilligen, G. Abbiendi, C. Battilana, D. Bonacorsi, L. Borgonovi, S. Braibant-Giacomelli, R. Campanini, P. Capiluppi, A. Castro, F. R. Cavallo, C. Ciocca, G. Codispoti, M. Cuffiani, G. M. Dallavalle, F. Fabbri, A. Fanfani, E. Fontanesi, P. Giacomelli, C. Grandi, L. Guiducci, F. Iemmi, S. Lo Meo, S. Marcellini, G. Masetti, F. L. Navarria, A. Perrotta, F. Primavera, A. M. Rossi, T. Rovelli, G. P. Siroli, N. Tosi, S. Albergo, S. Costa, A. Di Mattia, R. Potenza, A. Tricomi, C. Tuve, G. Barbagli, A. Cassese, R. Ceccarelli, V. Ciulli, C. Civinini, R. D’Alessandro, E. Focardi, G. Latino, P. Lenzi, M. Meschini, S. Paoletti, G. Sguazzoni, L. Viliani, L. Benussi, S. Bianco, D. Piccolo, M. Bozzo, F. Ferro, R. Mulargia, E. Robutti, S. Tosi, A. Benaglia, A. Beschi, F. Brivio, V. Ciriolo, S. Di Guida, M. E. Dinardo, P. Dini, S. Gennai, A. Ghezzi, P. Govoni, L. Guzzi, M. Malberti, S. Malvezzi, D. Menasce, F. Monti, L. Moroni, M. Paganoni, D. Pedrini, S. Ragazzi, T. Tabarelli de Fatis, D. Zuolo, S. Buontempo, N. Cavallo, A. De Iorio, A. Di Crescenzo, F. Fabozzi, F. Fienga, G. Galati, A. O. M. Iorio, L. Lista, S. Meola, P. Paolucci, B. Rossi, C. Sciacca, E. Voevodina, P. Azzi, N. Bacchetta, D. D.Bisello, A. Boletti, A. Bragagnolo, R. Carlin, P. Checchia, P. De Castro Manzano, T. Dorigo, U. Dosselli, F. Gasparini, U. Gasparini, A. Gozzelino, S. Y. Hoh, P. Lujan, M. Margoni, A. T. Meneguzzo, J. Pazzini, M. Presilla, P. Ronchese, R. Rossin, F. Simonetto, A. Tiko, M. Tosi, M. Zanetti, P. Zotto, G. Zumerle, A. Braghieri, D. Fiorina, P. Montagna, S. P. Ratti, V. Re, M. Ressegotti, C. Riccardi, P. Salvini, I. Vai, P. Vitulo, M. Biasini, G. M. Bilei, D. Ciangottini, L. Fanò, P. Lariccia, R. Leonardi, G. Mantovani, V. Mariani, M. Menichelli, A. Rossi, A. Santocchia, D. Spiga, K. Androsov, P. Azzurri, G. Bagliesi, V. Bertacchi, L. Bianchini, T. Boccali, R. Castaldi, M. A. Ciocci, R. Dell’Orso, G. Fedi, L. Giannini, A. Giassi, M. T. Grippo, F. Ligabue, E. Manca, G. Mandorli, A. Messineo, F. Palla, A. Rizzi, G. Rolandi, S. Roy Chowdhury, A. Scribano, P. Spagnolo, R. Tenchini, G. Tonelli, N. Turini, A. Venturi, P. G. Verdini, F. Cavallari, M. Cipriani, D. Del Re, E. Di Marco, M. Diemoz, E. Longo, B. Marzocchi, P. Meridiani, G. Organtini, F. Pandolfi, R. Paramatti, C. Quaranta, S. Rahatlou, C. Rovelli, F. Santanastasio, L. Soffi, N. Amapane, R. Arcidiacono, S. Argiro, M. Arneodo, N. Bartosik, R. Bellan, A. Bellora, C. Biino, A. Cappati, N. Cartiglia, S. Cometti, M. Costa, R. Covarelli, N. Demaria, B. Kiani, C. Mariotti, S. Maselli, E. Migliore, V. Monaco, E. Monteil, M. Monteno, M. M. Obertino, G. Ortona, L. Pacher, N. Pastrone, M. Pelliccioni, G. L. Pinna Angioni, A. Romero, M. Ruspa, R. Salvatico, V. Sola, A. Solano, D. Soldi, A. Staiano, S. Belforte, V. Candelise, M. Casarsa, F. Cossutti, A. Da Rold, G. Della Ricca, F. Vazzoler, A. Zanetti, B. Kim, D. H. Kim, G. N. Kim, J. Lee, S. W. Lee, C. S. Moon, Y. D. Oh, S. I. Pak, S. Sekmen, D. C. Son, Y. C. Yang, H. Kim, D. H. Moon, G. Oh, B. Francois, T. J. Kim, J. Park, S. Cho, S. Choi, Y. Go, D. Gyun, S. Ha, B. Hong, K. Lee, K. S. Lee, J. Lim, J. Park, S. K. Park, Y. Roh, J. Yoo, J. Goh, H. S. Kim, J. Almond, J. H. Bhyun, J. Choi, S. Jeon, J. Kim, J. S. Kim, H. Lee, K. Lee, S. Lee, K. Nam, M. Oh, S. B. Oh, B. C. Radburn-Smith, U. K. Yang, H. D. Yoo, I. Yoon, G. B. Yu, D. Jeon, H. Kim, J. H. Kim, J. S. H. Lee, I. C. Park, I. J Watson, Y. Choi, C. Hwang, Y. Jeong, J. Lee, Y. Lee, I. Yu, V. Veckalns, V. Dudenas, A. Juodagalvis, G. Tamulaitis, J. Vaitkus, Z. A. Ibrahim, F. Mohamad Idris, W. A. T. Wan Abdullah, M. N. Yusli, Z. Zolkapli, J. F. Benitez, A. Castaneda Hernandez, J. A. Murillo Quijada, L. Valencia Palomo, H. Castilla-Valdez, E. De La Cruz-Burelo, I. Heredia-De La Cruz, R. Lopez-Fernandez, A. Sanchez-Hernandez, S. Carrillo Moreno, C. Oropeza Barrera, M. Ramirez-Garcia, F. Vazquez Valencia, J. Eysermans, I. Pedraza, H. A. Salazar Ibarguen, C. Uribe Estrada, A. Morelos Pineda, J. Mijuskovic, N. Raicevic, D. Krofcheck, S. Bheesette, P. H. Butler, A. Ahmad, M. Ahmad, Q. Hassan, H. R. Hoorani, W. A. Khan, M. A. Shah, M. Shoaib, M. Waqas, V. Avati, L. Grzanka, M. Malawski, H. Bialkowska, M. Bluj, B. Boimska, M. Górski, M. Kazana, M. Szleper, P. Zalewski, K. Bunkowski, A. Byszuk, K. Doroba, A. Kalinowski, M. Konecki, J. Krolikowski, M. Misiura, M. Olszewski, M. Walczak, M. Araujo, P. Bargassa, D. Bastos, A. Di Francesco, P. Faccioli, B. Galinhas, M. Gallinaro, J. Hollar, N. Leonardo, J. Seixas, K. Shchelina, G. Strong, O. Toldaiev, J. Varela, S. Afanasiev, P. Bunin, M. Gavrilenko, I. Golutvin, I. Gorbunov, A. Kamenev, V. Karjavine, A. Lanev, A. Malakhov, V. Matveev, P. Moisenz, V. Palichik, V. Perelygin, M. Savina, S. Shmatov, S. Shulha, N. Skatchkov, V. Smirnov, N. Voytishin, A. Zarubin, L. Chtchipounov, V. Golovtcov, Y. Ivanov, V. Kim, E. Kuznetsova, P. Levchenko, V. Murzin, V. Oreshkin, I. Smirnov, D. Sosnov, V. Sulimov, L. Uvarov, A. Vorobyev, Yu. Andreev, A. Dermenev, S. Gninenko, N. Golubev, A. Karneyeu, M. Kirsanov, N. Krasnikov, A. Pashenkov, D. Tlisov, A. Toropin, V. Epshteyn, V. Gavrilov, N. Lychkovskaya, A. Nikitenko, V. Popov, I. Pozdnyakov, G. Safronov, A. Spiridonov, A. Stepennov, M. Toms, E. Vlasov, A. Zhokin, T. Aushev, M. Chadeeva, P. Parygin, D. Philippov, E. Popova, V. Rusinov, V. Andreev, M. Azarkin, I. Dremin, M. Kirakosyan, A. Terkulov, A. Baskakov, A. Belyaev, E. Boos, V. Bunichev, M. Dubinin, L. Dudko, A. Gribushin, A. Ershov, A. Gribushin, V. Klyukhin, N. Korneeva, I. Lokhtin, S. Obraztsov, M. Perfilov, V. Savrin, A. Barnyakov, V. Blinov, T. Dimova, L. Kardapoltsev, Y. Skovpen, I. Azhgirey, I. Bayshev, S. Bitioukov, V. Kachanov, D. Konstantinov, P. Mandrik, V. Petrov, R. Ryutin, S. Slabospitskii, A. Sobol, S. Troshin, N. Tyurin, A. Uzunian, A. Volkov, A. Babaev, A. Iuzhakov, V. Okhotnikov, V. Borchsh, V. Ivanchenko, E. Tcherniaev, P. Adzic, P. Cirkovic, D. Devetak, M. Dordevic, P. Milenovic, J. Milosevic, M. Stojanovic, M. Aguilar-Benitez, J. Alcaraz Maestre, A. lvarez Fernández, I. Bachiller, M. Barrio Luna, J. A. Brochero Cifuentes, C. A. Carrillo Montoya, M. Cepeda, M. Cerrada, N. Colino, B. De La Cruz, A. Delgado Peris, C. Fernandez Bedoya, J. P. Fernández Ramos, J. Flix, M. C. Fouz, O. Gonzalez Lopez, S. Goy Lopez, J. M. Hernandez, M. I. Josa, D. Moran, Navarro Tobar, A. Pérez-Calero Yzquierdo, J. Puerta Pelayo, I. Redondo, L. Romero, S. Sánchez Navas, M. S. Soares, A. Triossi, C. Willmott, C. Albajar, J. F. de Trocóniz, R. Reyes-Almanza, B. Alvarez Gonzalez, J. Cuevas, C. Erice, J. Fernandez Menendez, S. Folgueras, I. Gonzalez Caballero, J. R. González Fernández, E. Palencia Cortezon, V. Rodríguez Bouza, S. Sanchez Cruz, I. J. Cabrillo, A. Calderon, B. Chazin Quero, J. Duarte Campderros, M. Fernandez, P. J. Fernández Manteca, A. García Alonso, G. Gomez, C. Martinez Rivero, P. Martinez Ruiz del Arbol, F. Matorras, J. Piedra Gomez, C. Prieels, T. Rodrigo, A. Ruiz-Jimeno, L. Russo, L. Scodellaro, N. Trevisani, I. Vila, J. M. Vizan Garcia, K. Malagalage, W. G. D. Dharmaratna, N. Wickramage, D. Abbaneo, B. Akgun, E. Auffray, G. Auzinger, J. Baechler, P. Baillon, A. H. Ball, D. Barney, J. Bendavid, M. Bianco, A. Bocci, P. Bortignon, E. Bossini, C. Botta, E. Brondolin, T. Camporesi, A. Caratelli, G. Cerminara, E. Chapon, G. Cucciati, D. d’Enterria, A. Dabrowski, N. Daci, V. Daponte, A. David, O. Davignon, A. De Roeck, N. Deelen, M. Deile, M. Dobson, M. Dünser, N. Dupont, A. Elliott-Peisert, N. Emriskova, F. Fallavollita, D. Fasanella, S. Fiorendi, G. Franzoni, J. Fulcher, W. Funk, S. Giani, D. Gigi, A. Gilbert, K. Gill, F. Glege, M. Gruchala, M. Guilbaud, D. Gulhan, J. Hegeman, C. Heidegger, Y. Iiyama, V. Innocente, P. Janot, O. Karacheban, J. Kaspar, J. Kieseler, M. Krammer, N. Kratochwil, C. Lange, P. Lecoq, C. Lourenço, L. Malgeri, M. Mannelli, A. Massironi, F. Meijers, J. A. Merlin, S. Mersi, E. Meschi, F. Moortgat, M. Mulders, J. Ngadiuba, J. Niedziela, S. Nourbakhsh, S. Orfanelli, L. Orsini, F. Pantaleo, L. Pape, E. Perez, M. Peruzzi, A. Petrilli, G. Petrucciani, A. Pfeiffer, M. Pierini, F. M. Pitters, D. Rabady, A. Racz, M. Rieger, M. Rovere, H. Sakulin, C. Schäfer, C. Schwick, M. Selvaggi, A. Sharma, P. Silva, W. Snoeys, P. Sphicas, J. Steggemann, S. Summers, V. R. Tavolaro, D. Treille, A. Tsirou, G. P. Van Onsem, A. Vartak, M. Verzetti, W. D. Zeuner, L. Caminada, K. Deiters, W. Erdmann, R. Horisberger, Q. Ingram, H. C. Kaestli, D. Kotlinski, U. Langenegger, T. Rohe, S. A. Wiederkehr, M. Backhaus, P. Berger, N. Chernyavskaya, G. Dissertori, M. Dittmar, M. Donegà, C. Dorfer, T. A. Gómez Espinosa, C. Grab, D. Hits, T. Klijnsma, W. Lustermann, R. A. Manzoni, M. Marionneau, M. T. Meinhard, F. Micheli, P. Musella, F. Nessi-Tedaldi, F. Pauss, G. Perrin, L. Perrozzi, S. Pigazzini, M. G. Ratti, M. Reichmann, C. Reissel, T. Reitenspiess, D. Ruini, D. A. Sanz Becerra, M. Schönenberger, L. Shchutska, M. L. Vesterbacka Olsson, R. Wallny, D. H. Zhu, T. K. Aarrestad, C. Amsler, D. Brzhechko, M. F. Canelli, A. De Cosa, R. Del Burgo, S. Donato, B. Kilminster, S. Leontsinis, V. M. Mikuni, I. Neutelings, G. Rauco, P. Robmann, D. Salerno, K. Schweiger, C. Seitz, Y. Takahashi, S. Wertz, A. Zucchetta, T. H. Doan, C. M. Kuo, W. Lin, A. Roy, S. S. Yu, P. Chang, Y. Chao, K. F. Chen, P. H. Chen, W.-S. Hou, Y. y. Li, R.-S. Lu, E. Paganis, A. Psallidas, A. Steen, B. Asavapibhop, C. Asawatangtrakuldee, N. Srimanobhas, N. Suwonjandee, A. Bat, F. Boran, A. Celik, S. Cerci, S. Damarseckin, Z. S. Demiroglu, F. Dolek, C. Dozen, I. Dumanoglu, G. Gokbulut, EmineGurpinar Guler, Y. Guler, I. Hos, C. Isik, E. E. Kangal, O. Kara, A. Kayis Topaksu, U. Kiminsu, G. Onengut, K. Ozdemir, S. Ozturk, A. E. Simsek, D. Sunar Cerci, U. G. Tok, S. Turkcapar, I. S. Zorbakir, C. Zorbilmez, B. Isildak, G. Karapinar, M. Yalvac, I. O. Atakisi, E. Gülmez, M. Kaya, O. Kaya, Ö. Özçelik, S. Tekten, E. A. Yetkin, A. Cakir, K. Cankocak, Y. Komurcu, S. Sen, B. Kaynak, S. Ozkorucuklu, B. Grynyov, L. Levchuk, E. Bhal, S. Bologna, J. J. Brooke, D. Burns, E. Clement, D. Cussans, H. Flacher, J. Goldstein, G. P. Heath, H. F. Heath, L. Kreczko, S. Paramesvaran, B. Penning, T. Sakuma, S. Seif El Nasr-Storey, V. J. Smith, J. Taylor, A. Titterton, K. W. Bell, A. Belyaev, C. Brew, R. M. Brown, D. Cieri, D. J. A. Cockerill, J. A. Coughlan, K. Harder, S. Harper, J. Linacre, K. Manolopoulos, D. M. Newbold, E. Olaiya, D. Petyt, T. Reis, T. Schuh, C. H. Shepherd-Themistocleous, A. Thea, I. R. Tomalin, T. Williams, W. J. Womersley, R. Bainbridge, P. Bloch, J. Borg, S. Breeze, O. Buchmuller, A. Bundock, GurpreetSingh CHAHAL, D. Colling, P. Dauncey, G. Davies, M. Della Negra, R. Di Maria, P. Everaerts, G. Hall, G. Iles, T. James, M. Komm, C. Laner, L. Lyons, A.-M. Magnan, S. Malik, A. Martelli, V. Milosevic, J. Nash, V. Palladino, M. Pesaresi, D. M. Raymond, A. Richards, A. Rose, E. Scott, C. Seez, A. Shtipliyski, M. Stoye, T. Strebler, A. Tapper, K. Uchida, T. Virdee, N. Wardle, D. Winterbottom, J. Wright, A. G. Zecchinelli, S. C. Zenz, J. E. Cole, P. R. Hobson, A. Khan, P. Kyberd, C. K. Mackay, A. Morton, I. D. Reid, L. Teodorescu, S. Zahid, K. Call, B. Caraway, J. Dittmann, K. Hatakeyama, C. Madrid, B. McMaster, N. Pastika, C. Smith, R. Bartek, A. Dominguez, R. Uniyal, A. M. Vargas Hernandez, A. Buccilli, S. I. Cooper, C. Henderson, P. Rumerio, C. West, D. Arcaro, Z. Demiragli, D. Gastler, D. Pinna, C. Richardson, J. Rohlf, D. Sperka, I. Suarez, L. Sulak, D. Zou, G. Benelli, B. Burkle, X. Coubez, D. Cutts, Y. t. Duh, M. Hadley, J. Hakala, U. Heintz, J. M. Hogan, K. H. M. Kwok, E. Laird, G. Landsberg, J. Lee, Z. Mao, M. Narain, S. Sagir, R. Syarif, E. Usai, D. Yu, W. Zhang, R. Band, C. Brainerd, R. Breedon, M. Calderon De La Barca Sanchez, M. Chertok, J. Conway, R. Conway, P. T. Cox, R. Erbacher, C. Flores, G. Funk, F. Jensen, W. Ko, O. Kukral, R. Lander, M. Mulhearn, D. Pellett, J. Pilot, M. Shi, D. Taylor, K. Tos, M. Tripathi, Z. Wang, F. Zhang, M. Bachtis, C. Bravo, R. Cousins, A. Dasgupta, A. Florent, J. Hauser, M. Ignatenko, N. Mccoll, W. A. Nash, S. Regnard, D. Saltzberg, C. Schnaible, B. Stone, V. Valuev, K. Burt, Y. Chen, R. Clare, J. W. Gary, S. M. A. Ghiasi Shirazi, G. Hanson, G. Karapostoli, E. Kennedy, O. R. Long, M. Olmedo Negrete, M. I. Paneva, W. Si, L. Wang, S. Wimpenny, B. R. Yates, Y. Zhang, J. G. Branson, P. Chang, S. Cittolin, M. Derdzinski, R. Gerosa, D. Gilbert, B. Hashemi, D. Klein, V. Krutelyov, J. Letts, M. Masciovecchio, S. May, S. Padhi, M. Pieri, V. Sharma, M. Tadel, F. Würthwein, A. Yagil, G. Zevi Della Porta, N. Amin, R. Bhandari, C. Campagnari, M. Citron, V. Dutta, M. Franco Sevilla, L. Gouskos, J. Incandela, B. Marsh, H. Mei, A. Ovcharova, H. Qu, J. Richman, U. Sarica, D. Stuart, S. Wang, D. Anderson, A. Bornheim, O. Cerri, I. Dutta, J. M. Lawhorn, N. Lu, J. Mao, H. B. Newman, T. Q. Nguyen, J. Pata, M. Spiropulu, J. R. Vlimant, S. Xie, Z. Zhang, R. Y. Zhu, M. B. Andrews, T. Ferguson, T. Mudholkar, M. Paulini, M. Sun, I. Vorobiev, M. Weinberg, J. P. Cumalat, W. T. Ford, A. Johnson, E. MacDonald, T. Mulholland, R. Patel, A. Perloff, K. Stenson, K. A. Ulmer, S. R. Wagner, J. Alexander, J. Chaves, Y. Cheng, J. Chu, A. Datta, A. Frankenthal, K. Mcdermott, J. R. Patterson, D. Quach, A. Rinkevicius, A. Ryd, S. M. Tan, Z. Tao, J. Thom, P. Wittich, M. Zientek, S. Abdullin, M. Albrow, M. Alyari, G. Apollinari, A. Apresyan, A. Apyan, S. Banerjee, L. A. T. Bauerdick, A. Beretvas, D. Berry, J. Berryhill, P. C. Bhat, K. Burkett, J. N. Butler, A. Canepa, G. B. Cerati, H. W. K. Cheung, F. Chlebana, M. Cremonesi, J. Duarte, V. D. Elvira, J. Freeman, Z. Gecse, E. Gottschalk, L. Gray, D. Green, S. Grünendahl, O. Gutsche, AllisonReinsvold Hall, J. Hanlon, R. M. Harris, S. Hasegawa, R. Heller, J. Hirschauer, B. Jayatilaka, S. Jindariani, M. Johnson, U. Joshi, B. Klima, M. J. Kortelainen, B. Kreis, S. Lammel, J. Lewis, D. Lincoln, R. Lipton, M. Liu, T. Liu, J. Lykken, K. Maeshima, J. M. Marraffino, D. Mason, P. McBride, P. Merkel, S. Mrenna, S. Nahn, V. O’Dell, V. Papadimitriou, K. Pedro, C. Pena, G. Rakness, F. Ravera, L. Ristori, B. Schneider, E. Sexton-Kennedy, N. Smith, A. Soha, W. J. Spalding, L. Spiegel, S. Stoynev, J. Strait, N. Strobbe, L. Taylor, S. Tkaczyk, N. V. Tran, L. Uplegger, E. W. Vaandering, C. Vernieri, R. Vidal, M. Wang, H. A. Weber, D. Acosta, P. Avery, D. Bourilkov, A. Brinkerhoff, L. Cadamuro, A. Carnes, V. Cherepanov, F. Errico, R. D. Field, S. V. Gleyzer, B. M. Joshi, M. Kim, J. Konigsberg, A. Korytov, K. H. Lo, P. Ma, K. Matchev, N. Menendez, G. Mitselmakher, D. Rosenzweig, K. Shi, J. Wang, S. Wang, X. Zuo, Y. R. Joshi, T. Adams, A. Askew, S. Hagopian, V. Hagopian, K. F. Johnson, R. Khurana, T. Kolberg, G. Martinez, T. Perry, H. Prosper, C. Schiber, R. Yohay, J. Zhang, M. M. Baarmand, M. Hohlmann, D. Noonan, M. Rahmani, M. Saunders, F. Yumiceva, M. R. Adams, L. Apanasevich, R. R. Betts, R. Cavanaugh, X. Chen, S. Dittmer, O. Evdokimov, C. E. Gerber, D. A. Hangal, D. J. Hofman, K. Jung, C. Mills, T. Roy, M. B. Tonjes, N. Varelas, J. Viinikainen, H. Wang, X. Wang, Z. Wu, M. Alhusseini, B. Bilki, W. Clarida, K. Dilsiz, S. Durgut, R. P. Gandrajula, M. Haytmyradov, V. Khristenko, O. K. Köseyan, J.-P. Merlo, A. Mestvirishvili, A. Moeller, J. Nachtman, H. Ogul, Y. Onel, F. Ozok, A. Penzo, C. Snyder, E. Tiras, J. Wetzel, B. Blumenfeld, A. Cocoros, N. Eminizer, A. V. Gritsan, W. T. Hung, P. Maksimovic, J. Roskes, M. Swartz, C. Baldenegro Barrera, P. Baringer, A. Bean, S. Boren, J. Bowen, A. Bylinkin, T. Isidori, S. Khalil, J. King, G. Krintiras, A. Kropivnitskaya, C. Lindsey, D. Majumder, W. Mcbrayer, N. Minafra, M. Murray, C. Rogan, C. Royon, S. Sanders, E. Schmitz, J. D. Tapia Takaki, Q. Wang, J. Williams, G. Wilson, S. Duric, A. Ivanov, K. Kaadze, D. Kim, Y. Maravin, D. R. Mendis, T. Mitchell, A. Modak, A. Mohammadi, F. Rebassoo, D. Wright, A. Baden, O. Baron, A. Belloni, S. C. Eno, Y. Feng, N. J. Hadley, S. Jabeen, G. Y. Jeng, R. G. Kellogg, J. Kunkle, A. C. Mignerey, S. Nabili, F. Ricci-Tam, M. Seidel, Y. H. Shin, A. Skuja, S. C. Tonwar, K. Wong, D. Abercrombie, B. Allen, A. Baty, R. Bi, S. Brandt, W. Busza, I. A. Cali, M. D’Alfonso, G. Gomez Ceballos, M. Goncharov, P. Harris, D. Hsu, M. Hu, M. Klute, D. Kovalskyi, Y.-J. Lee, P. D. Luckey, B. Maier, A. C. Marini, C. Mcginn, C. Mironov, S. Narayanan, X. Niu, C. Paus, D. Rankin, C. Roland, G. Roland, Z. Shi, G. S. F. Stephans, K. Sumorok, K. Tatar, D. Velicanu, J. Wang, T. W. Wang, B. Wyslouch, R. M. Chatterjee, A. Evans, S. Guts, P. Hansen, J. Hiltbrand, Sh. Jain, Y. Kubota, Z. Lesko, J. Mans, R. Rusack, M. A. Wadud, J. G. Acosta, S. Oliveros, K. Bloom, D. R. Claes, C. Fangmeier, L. Finco, F. Golf, R. Kamalieddin, I. Kravchenko, J. E. Siado, G. R. Snow, B. Stieger, W. Tabb, G. Agarwal, C. Harrington, I. Iashvili, A. Kharchilava, C. McLean, D. Nguyen, A. Parker, J. Pekkanen, S. Rappoccio, B. Roozbahani, G. Alverson, E. Barberis, C. Freer, Y. Haddad, A. Hortiangtham, G. Madigan, D. M. Morse, T. Orimoto, L. Skinnari, A. Tishelman-Charny, T. Wamorkar, B. Wang, A. Wisecarver, D. Wood, S. Bhattacharya, J. Bueghly, T. Gunter, K. A. Hahn, N. Odell, M. H. Schmitt, K. Sung, M. Trovato, M. Velasco, R. Bucci, N. Dev, R. Goldouzian, M. Hildreth, K. Hurtado Anampa, C. Jessop, D. J. Karmgard, K. Lannon, W. Li, N. Loukas, N. Marinelli, I. Mcalister, F. Meng, C. Mueller, Y. Musienko, M. Planer, R. Ruchti, P. Siddireddy, G. Smith, S. Taroni, M. Wayne, A. Wightman, M. Wolf, A. Woodard, J. Alimena, B. Bylsma, L. S. Durkin, S. Flowers, B. Francis, C. Hill, W. Ji, A. Lefeld, T. Y. Ling, B. L. Winer, S. Cooperstein, G. Dezoort, P. Elmer, J. Hardenbrook, N. Haubrich, S. Higginbotham, A. Kalogeropoulos, S. Kwan, D. Lange, M. T. Lucchini, J. Luo, D. Marlow, K. Mei, I. Ojalvo, J. Olsen, C. Palmer, P. Piroué, J. Salfeld-Nebgen, D. Stickland, C. Tully, Z. Wang, S. Malik, S. Norberg, A. Barker, V. E. Barnes, S. Das, L. Gutay, M. Jones, A. W. Jung, A. Khatiwada, B. Mahakud, D. H. Miller, G. Negro, N. Neumeister, C. C. Peng, S. Piperov, H. Qiu, J. F. Schulte, J. Sun, F. Wang, R. Xiao, W. Xie, T. Cheng, J. Dolen, N. Parashar, U. Behrens, K. M. Ecklund, S. Freed, F. J. M. Geurts, M. Kilpatrick, Arun Kumar, W. Li, B. P. Padley, R. Redjimi, J. Roberts, J. Rorie, W. Shi, A. G. Stahl Leiton, Z. Tu, A. Zhang, A. Bodek, P. de Barbaro, R. Demina, J. L. Dulemba, C. Fallon, T. Ferbel, M. Galanti, A. Garcia-Bellido, O. Hindrichs, A. Khukhunaishvili, E. Ranken, R. Taus, B. Chiarito, J. P. Chou, A. Gandrakota, Y. Gershtein, E. Halkiadakis, A. Hart, M. Heindl, E. Hughes, S. Kaplan, S. Kyriacou, I. Laflotte, A. Lath, R. Montalvo, K. Nash, M. Osherson, H. Saka, S. Salur, S. Schnetzer, S. Somalwar, R. Stone, S. Thomas, H. Acharya, A. G. Delannoy, G. Riley, S. Spanier, O. Bouhali, A. Celik, M. Dalchenko, M. De Mattia, A. Delgado, S. Dildick, R. Eusebi, J. Gilmore, T. Huang, T. Kamon, S. Luo, D. Marley, R. Mueller, D. Overton, L. Perniè, D. Rathjens, A. Safonov, N. Akchurin, J. Damgov, F. De Guio, S. Kunori, K. Lamichhane, S. W. Lee, T. Mengke, S. Muthumuni, T. Peltola, S. Undleeb, I. Volobouev, Z. Wang, A. Whitbeck, S. Greene, A. Gurrola, R. Janjam, W. Johns, C. Maguire, A. Melo, H. Ni, K. Padeken, F. Romeo, P. Sheldon, S. Tuo, J. Velkovska, M. Verweij, M. W. Arenton, P. Barria, B. Cox, G. Cummings, R. Hirosky, M. Joyce, A. Ledovskoy, C. Neu, B. Tannenwald, Y. Wang, E. Wolfe, F. Xia, R. Harr, P. E. Karchin, N. Poudyal, J. Sturdy, P. Thapa, T. Bose, J. Buchanan, C. Caillol, D. Carlsmith, S. Dasu, I. De Bruyn, L. Dodd, F. Fiori, C. Galloni, B. Gomber, H. He, M. Herndon, A. Hervé, U. Hussain, P. Klabbers, A. Lanaro, A. Loeliger, K. Long, R. Loveless, J. Madhusudanan Sreekala, T. Ruggles, A. Savin, V. Sharma, W. H. Smith, D. Teague, S. Trembath-reichert, N. Woods, CMS Collaboration

**Affiliations:** 1 Yerevan Physics Institute, Yerevan, Armenia; 2 Institut für Hochenergiephysik, Wien, Austria; 3 Institute for Nuclear Problems, Minsk, Belarus; 4 Universiteit Antwerpen, Antwerp, Belgium; 5 Vrije Universiteit Brussel, Brussels, Belgium; 6 Université Libre de Bruxelles, Brussels, Belgium; 7 Ghent University, Ghent, Belgium; 8 Université Catholique de Louvain, Louvain-la-Neuve, Belgium; 9 Centro Brasileiro de Pesquisas Fisicas, Rio de Janeiro, Brazil; 10 Universidade do Estado do Rio de Janeiro, Rio de Janeiro, Brazil; 11 Universidade Estadual Paulista, Universidade Federal do ABC, São Paulo, Brazil; 12 Institute for Nuclear Research and Nuclear Energy, Bulgarian Academy of Sciences, Sofia, Bulgaria; 13 University of Sofia, Sofia, Bulgaria; 14 Beihang University, Beijing, China; 15 Institute of High Energy Physics, Beijing, China; 16 State Key Laboratory of Nuclear Physics and Technology, Peking University, Beijing, China; 17 Tsinghua University, Beijing, China; 18 Zhejiang University, Hangzhou, China; 19 Universidad de Los Andes, Bogotá, Colombia; 20 Universidad de Antioquia, Medellin, Colombia; 21 University of Split, Faculty of Electrical Engineering, Mechanical Engineering and Naval Architecture, Split, Croatia; 22 University of Split, Faculty of Science, Split, Croatia; 23 Institute Rudjer Boskovic, Zagreb, Croatia; 24 University of Cyprus, Nicosia, Cyprus; 25 Charles University, Prague, Czech Republic; 26 Escuela Politecnica Nacional, Quito, Ecuador; 27 Universidad San Francisco de Quito, Quito, Ecuador; 28 Academy of Scientific Research and Technology of the Arab Republic of Egypt, Egyptian Network of High Energy Physics, Cairo, Egypt; 29 National Institute of Chemical Physics and Biophysics, Tallinn, Estonia; 30 Department of Physics, University of Helsinki, Helsinki, Finland; 31 Helsinki Institute of Physics, Helsinki, Finland; 32 Lappeenranta University of Technology, Lappeenranta, Finland; 33 IRFU, CEA, Université Paris-Saclay, Gif-sur-Yvette, France; 34 Laboratoire Leprince-Ringuet, CNRS/IN2P3, Ecole Polytechnique, Institut Polytechnique de Paris, Palaiseau, France; 35 Université de Strasbourg, CNRS, IPHC UMR 7178, Strasbourg, France; 36 Centre de Calcul de l’Institut National de Physique Nucleaire et de Physique des Particules, CNRS/IN2P3, Villeurbanne, France; 37 Université de Lyon, Université Claude Bernard Lyon 1, CNRS-IN2P3, Institut de Physique Nucléaire de Lyon, Villeurbanne, France; 38 Georgian Technical University, Tbilisi, Georgia; 39 Tbilisi State University, Tbilisi, Georgia; 40 RWTH Aachen University, I. Physikalisches Institut, Aachen, Germany; 41 RWTH Aachen University, III. Physikalisches Institut A, Aachen, Germany; 42 RWTH Aachen University, III. Physikalisches Institut B, Aachen, Germany; 43 Deutsches Elektronen-Synchrotron, Hamburg, Germany; 44 University of Hamburg, Hamburg, Germany; 45 Karlsruher Institut fuer Technologie, Karlsruhe, Germany; 46 Institute of Nuclear and Particle Physics (INPP), NCSR Demokritos, Aghia Paraskevi, Greece; 47 National and Kapodistrian University of Athens, Athens, Greece; 48 National Technical University of Athens, Athens, Greece; 49 University of Ioánnina, Ioannina, Greece; 50 MTA-ELTE Lendület CMS Particle and Nuclear Physics Group, Eötvös Loránd University, Budapest, Hungary; 51 Wigner Research Centre for Physics, Budapest, Hungary; 52 Institute of Nuclear Research ATOMKI, Debrecen, Hungary; 53 Institute of Physics, University of Debrecen, Debrecen, Hungary; 54 Eszterhazy Karoly University, Karoly Robert Campus, Gyongyos, Hungary; 55 Indian Institute of Science (IISc), Bangalore, India; 56 National Institute of Science Education and Research, HBNI, Bhubaneswar, India; 57 Panjab University, Chandigarh, India; 58 University of Delhi, Delhi, India; 59 Saha Institute of Nuclear Physics, HBNI, Kolkata, India; 60 Indian Institute of Technology Madras, Chennai, India; 61 Bhabha Atomic Research Centre, Mumbai, India; 62 Tata Institute of Fundamental Research-A, Mumbai, India; 63 Tata Institute of Fundamental Research-B, Mumbai, India; 64 Indian Institute of Science Education and Research (IISER), Pune, India; 65 Institute for Research in Fundamental Sciences (IPM), Tehran, Iran; 66 University College Dublin, Dublin, Ireland; 67 INFN Sezione di Bari, Università di Bari, Politecnico di Bari, Bari, Italy; 68 INFN Sezione di Bologna, Università di Bologna, Bologna, Italy; 69 INFN Sezione di Catania, Università di Catania, Catania, Italy; 70 INFN Sezione di Firenze, Università di Firenze, Firenze, Italy; 71 INFN Laboratori Nazionali di Frascati, Frascati, Italy; 72 INFN Sezione di Genova, Università di Genova, Genoa, Italy; 73 INFN Sezione di Milano-Bicocca, Università di Milano-Bicocca, Milan, Italy; 74 INFN Sezione di Napoli, Università di Napoli ’Federico II’ , Napoli, Italy, Università della Basilicata, Potenza, Italy, Università G. Marconi, Rome, Italy; 75 INFN Sezione di Padova, Università di Padova, Padova, Italy, Università di Trento, Trento, Italy; 76 INFN Sezione di Pavia, Università di Pavia, Pavia, Italy; 77 INFN Sezione di Perugia, Università di Perugia, Perugia, Italy; 78 INFN Sezione di Pisa, Università di Pisa, Scuola Normale Superiore di Pisa, Pisa, Italy; 79 INFN Sezione di Roma, Sapienza Università di Roma, Rome, Italy; 80 INFN Sezione di Torino, Università di Torino, Torino, Italy, Università del Piemonte Orientale, Novara, Italy; 81 INFN Sezione di Trieste, Università di Trieste, Trieste, Italy; 82 Kyungpook National University, Daegu, Korea; 83 Chonnam National University, Institute for Universe and Elementary Particles, Kwangju, Korea; 84 Hanyang University, Seoul, Korea; 85 Korea University, Seoul, Korea; 86 Department of Physics, Kyung Hee University, Seoul, Korea; 87 Sejong University, Seoul, Korea; 88 Seoul National University, Seoul, Korea; 89 University of Seoul, Seoul, Korea; 90 Sungkyunkwan University, Suwon, Korea; 91 Riga Technical University, Riga, Latvia; 92 Vilnius University, Vilnius, Lithuania; 93 National Centre for Particle Physics, Universiti Malaya, Kuala Lumpur, Malaysia; 94 Universidad de Sonora (UNISON), Hermosillo, Mexico; 95 Centro de Investigacion y de Estudios Avanzados del IPN, Mexico City, Mexico; 96 Universidad Iberoamericana, Mexico City, Mexico; 97 Benemerita Universidad Autonoma de Puebla, Puebla, Mexico; 98 Universidad Autónoma de San Luis Potosí, San Luis Potosí, Mexico; 99 University of Montenegro, Podgorica, Montenegro; 100 University of Auckland, Auckland, New Zealand; 101 University of Canterbury, Christchurch, New Zealand; 102 National Centre for Physics, Quaid-I-Azam University, Islamabad, Pakistan; 103 AGH University of Science and Technology Faculty of Computer Science, Electronics and Telecommunications, Kraków, Poland; 104 National Centre for Nuclear Research, Swierk, Poland; 105 Institute of Experimental Physics, Faculty of Physics, University of Warsaw, Warsaw, Poland; 106 Laboratório de Instrumentação e Física Experimental de Partículas, Lisbon, Portugal; 107 Joint Institute for Nuclear Research, Dubna, Russia; 108 Petersburg Nuclear Physics Institute, Gatchina (St. Petersburg), Russia; 109 Institute for Nuclear Research, Moscow, Russia; 110 Institute for Theoretical and Experimental Physics named by A.I. Alikhanov of NRC ‘Kurchatov Institute’, Moscow, Russia; 111 Moscow Institute of Physics and Technology, Moscow, Russia; 112 National Research Nuclear University ’Moscow Engineering Physics Institute’ (MEPhI), Moscow, Russia; 113 P.N. Lebedev Physical Institute, Moscow, Russia; 114 Skobeltsyn Institute of Nuclear Physics, Lomonosov Moscow State University, Moscow, Russia; 115 Novosibirsk State University (NSU), Novosibirsk, Russia; 116 Institute for High Energy Physics of National Research Centre ‘Kurchatov Institute’, Protvino, Russia; 117 National Research Tomsk Polytechnic University, Tomsk, Russia; 118 Tomsk State University, Tomsk, Russia; 119 University of Belgrade: Faculty of Physics and VINCA Institute of Nuclear Sciences, Belgrad, Serbia; 120 Centro de Investigaciones Energéticas Medioambientales y Tecnológicas (CIEMAT), Madrid, Spain; 121 Universidad Autónoma de Madrid, Madrid, Spain; 122 Universidad de Oviedo, Instituto Universitario de Ciencias y Tecnologías Espaciales de Asturias (ICTEA), Oviedo, Spain; 123 Instituto de Física de Cantabria (IFCA), CSIC-Universidad de Cantabria, Santander, Spain; 124 University of Colombo, Colombo, Sri Lanka; 125 Department of Physics, University of Ruhuna, Matara, Sri Lanka; 126 CERN, European Organization for Nuclear Research, Geneva, Switzerland; 127 Paul Scherrer Institut, Villigen, Switzerland; 128 ETH Zurich-Institute for Particle Physics and Astrophysics (IPA), Zurich, Switzerland; 129 Universität Zürich, Zurich, Switzerland; 130 National Central University, Chung-Li, Taiwan; 131 National Taiwan University (NTU), Taipei, Taiwan; 132 Chulalongkorn University, Faculty of Science, Department of Physics, Bangkok, Thailand; 133 ukurova University, Physics Department, Science and Art Faculty, Adana, Turkey; 134 Middle East Technical University, Physics Department, Ankara, Turkey; 135 Bogazici University, Istanbul, Turkey; 136 Istanbul Technical University, Istanbul, Turkey; 137 Istanbul University, Istanbul, Turkey; 138 Institute for Scintillation Materials of National Academy of Science of Ukraine, Kharkov, Ukraine; 139 National Scientific Center, Kharkov Institute of Physics and Technology, Kharkov, Ukraine; 140 University of Bristol, Bristol, UK; 141 Rutherford Appleton Laboratory, Didcot, UK; 142 Imperial College, London, UK; 143 Brunel University, Uxbridge, UK; 144 Baylor University, Waco, USA; 145 Catholic University of America, Washington, DC USA; 146 The University of Alabama, Tuscaloosa, USA; 147 Boston University, Boston, USA; 148 Brown University, Providence, USA; 149 University of California, Davis, USA; 150 University of California, Los Angeles, USA; 151 University of California, Riverside, Riverside, USA; 152 University of California, San Diego, La Jolla, USA; 153 University of California, Santa Barbara-Department of Physics, Santa Barbara, USA; 154 California Institute of Technology, Pasadena, USA; 155 Carnegie Mellon University, Pittsburgh, USA; 156 University of Colorado Boulder, Boulder, USA; 157 Cornell University, Ithaca, USA; 158 Fermi National Accelerator Laboratory, Batavia, USA; 159 University of Florida, Gainesville, USA; 160 Florida International University, Miami, USA; 161 Florida State University, Tallahassee, USA; 162 Florida Institute of Technology, Melbourne, USA; 163 University of Illinois at Chicago (UIC), Chicago, USA; 164 The University of Iowa, Iowa City, USA; 165 Johns Hopkins University, Baltimore, USA; 166 The University of Kansas, Lawrence, USA; 167 Kansas State University, Manhattan, USA; 168 Lawrence Livermore National Laboratory, Livermore, USA; 169 University of Maryland, College Park, USA; 170 Massachusetts Institute of Technology, Cambridge, USA; 171 University of Minnesota, Minneapolis, USA; 172 University of Mississippi, Oxford, USA; 173 University of Nebraska-Lincoln, Lincoln, USA; 174 State University of New York at Buffalo, Buffalo, USA; 175 Northeastern University, Boston, USA; 176 Northwestern University, Evanston, USA; 177 University of Notre Dame, Notre Dame, USA; 178 The Ohio State University, Columbus, USA; 179 Princeton University, Princeton, USA; 180 University of Puerto Rico, Mayaguez, USA; 181 Purdue University, West Lafayette, USA; 182 Purdue University Northwest, Hammond, USA; 183 Rice University, Houston, USA; 184 University of Rochester, Rochester, USA; 185 Rutgers, The State University of New Jersey, Piscataway, USA; 186 University of Tennessee, Knoxville, USA; 187 Texas A & M University, College Station, USA; 188 Texas Tech University, Lubbock, USA; 189 Vanderbilt University, Nashville, USA; 190 University of Virginia, Charlottesville, USA; 191 Wayne State University, Detroit, USA; 192 University of Wisconsin-Madison, Madison, WI USA

## Abstract

The standard model (SM) production of four top quarks ($\text {t} {}{\overline{\text {t}}} \text {t} {}{\overline{\text {t}}}$) in proton–proton collisions is studied by the CMS Collaboration. The data sample, collected during the 2016–2018 data taking of the LHC, corresponds to an integrated luminosity of 137$\,\text {fb}^{-1}$ at a center-of-mass energy of 13$\,\text {TeV}$. The events are required to contain two same-sign charged leptons (electrons or muons) or at least three leptons, and jets. The observed and expected significances for the $\text {t} {}{\overline{\text {t}}} \text {t} {}{\overline{\text {t}}}$ signal are respectively 2.6 and 2.7 standard deviations, and the $\text {t} {}{\overline{\text {t}}} \text {t} {}{\overline{\text {t}}}$ cross section is measured to be $12.6^{+5.8}_{-5.2}\,\text {fb}$. The results are used to constrain the Yukawa coupling of the top quark to the Higgs boson, $y_{\text {t}}$, yielding a limit of $|y_{\text {t}}/y_{\text {t}}^{\mathrm {SM}} | < 1.7$ at $95\%$ confidence level, where $y_{\text {t}}^{\mathrm {SM}}$ is the SM value of $y_{\text {t}}$. They are also used to constrain the oblique parameter of the Higgs boson in an effective field theory framework, $\hat{H}<0.12$. Limits are set on the production of a heavy scalar or pseudoscalar boson in Type-II two-Higgs-doublet and simplified dark matter models, with exclusion limits reaching 350–470$\,\text {GeV}$ and 350–550$\,\text {GeV}$ for scalar and pseudoscalar bosons, respectively. Upper bounds are also set on couplings of the top quark to new light particles.

## Introduction

**Fig. 1:**
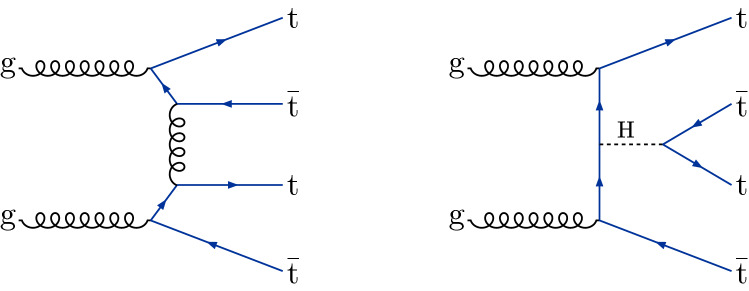
Typical Feynman diagrams for $\text {t} {}{\overline{\text {t}}} \text {t} {}{\overline{\text {t}}}$ production at leading order in the SM

The production of four top quarks ($\text {t} {}{\overline{\text {t}}} \text {t} {}{\overline{\text {t}}}$) is a rare standard model (SM) process, with a predicted cross section of $\sigma (\hbox {pp} \rightarrow \text {t} {}{\overline{\text {t}}} \text {t} {}{\overline{\text {t}}} ) = 12.0^{+2.2}_{-2.5}\,\text {fb}$ in proton–proton ($\hbox {pp}$) collisions at a center-of-mass energy of 13$\,\text {TeV}$, as calculated at next-to-leading-order (NLO) accuracy for both quantum chromodynamics and electroweak interactions [1]. Representative leading-order (LO) Feynman diagrams for SM production of $\text {t} {}{\overline{\text {t}}} \text {t} {}{\overline{\text {t}}}$ are shown in Fig. 1.

The $\text {t} {}{\overline{\text {t}}} \text {t} {}{\overline{\text {t}}}$ cross section can be used to constrain the magnitude and CP properties of the Yukawa coupling of the top quark to the Higgs boson [2, 3]. Moreover, $\text {t} {}{\overline{\text {t}}} \text {t} {}{\overline{\text {t}}}$ production can be significantly enhanced by beyond-the-SM (BSM) particles and interactions. New particles coupled to the top quark, such as heavy scalar and pseudoscalar bosons predicted in Type-II two-Higgs-doublet models (2HDM) [4–6] and by simplified models of dark matter (DM) [7, 8], can contribute to $\sigma (\hbox {pp} \rightarrow \text {t} {}{\overline{\text {t}}} \text {t} {}{\overline{\text {t}}}$) when their masses are larger than twice the mass of the top quark, with diagrams similar to Fig. 1 (right). Additionally, less massive particles can enhance $\sigma (\hbox {pp} \rightarrow \text {t} {}{\overline{\text {t}}} \text {t} {}{\overline{\text {t}}}$) via off-shell contributions [9]. In the model-independent framework of SM effective field theory, four-fermion couplings [10], as well as a modifier to the Higgs boson propagator [11], can be constrained through a measurement of $\sigma (\hbox {pp} \rightarrow \text {t} {}{\overline{\text {t}}} \text {t} {}{\overline{\text {t}}}$). Conversely, models with new particles with masses on the order of 1$\,\text {TeV}$, such as gluino pair production in the framework of supersymmetry [12–21], are more effectively probed through studies of $\text {t} {}{\overline{\text {t}}} \text {t} {}{\overline{\text {t}}}$ production in boosted events or by requiring very large imbalances in momentum.

Each top quark primarily decays to a bottom quark and a W boson, and each W boson decays to either leptons or quarks. As a result, the $\text {t} {}{\overline{\text {t}}} \text {t} {}{\overline{\text {t}}}$ final state contains jets mainly from the hadronization of light (u, d, s, c) quarks (light-flavor jets) and b  quarks (b  jets), and can also contain isolated charged leptons and missing transverse momentum arising from emitted neutrinos. Final states with either two same-sign leptons or at least three leptons, considering $\hbox {W} \rightarrow \ell \nu$ ($\ell = \hbox {e}$ or $\mu$) and including leptonic decays of $\tau$ leptons, correspond to a combined branching fraction of approximately 12% [22]. The relatively low levels of background make these channels the most sensitive to $\text {t} {}{\overline{\text {t}}} \text {t} {}{\overline{\text {t}}}$ events produced with SM-like kinematic properties [23].

Previous searches for $\text {t} {}{\overline{\text {t}}} \text {t} {}{\overline{\text {t}}}$ production in 13$\,\text {TeV}$\,$\hbox {pp}$ collisions were performed by the ATLAS [24, 25] and CMS [23, 26, 27] Collaborations. The most sensitive results, based on an integrated luminosity of approximately $36{\,\text {fb}^{-1}}$ collected by each experiment, led to cross section measurements of $28.5^{+12}_{-11}\,\text {fb}$ with an observed (expected) significance of 2.8 (1.0) standard deviations by ATLAS [25], and $13^{+11}_{-9}\,\text {fb}$ with an observed (expected) significance of 1.4 (1.1) standard deviations by CMS [23], both consistent with the SM prediction.

The analysis described in this paper improves upon the CMS search presented in Ref. [27], and supersedes the results, by taking advantage of upgrades to the CMS detector and by optimizing the definitions of the signal regions for the integrated luminosity of 137$\,\text {fb}^{-1}$. The reference cross section for SM $\text {t} {}{\overline{\text {t}}} \text {t} {}{\overline{\text {t}}}$, $12.0^{+2.2}_{-2.5}\,\text {fb}$, used to determine the expected statistical significance of the search, as well as in interpretations for which SM $\text {t} {}{\overline{\text {t}}} \text {t} {}{\overline{\text {t}}}$ is a background, includes NLO electroweak effects, in contrast to the $9.2^{+2.9}_{-2.4}\,\text {fb}$ [28] used in the previous search. In addition to the analysis strategy used in the previous search, a new multivariate classifier is defined to maximize the sensitivity to the SM $\text {t} {}{\overline{\text {t}}} \text {t} {}{\overline{\text {t}}}$ signal.

## Background and signal simulation

Monte Carlo (MC) simulated samples at NLO are used to evaluate the signal acceptance for the SM $\text {t} {}{\overline{\text {t}}} \text {t} {}{\overline{\text {t}}}$ process and to estimate the backgrounds from diboson ($\hbox {WZ}$, $\hbox {ZZ}$, $\hbox {Z} \upgamma$, $\hbox {W} ^{\pm }\hbox {W} ^{\pm }$) and triboson ($\hbox {WWW}$, $\hbox {WWZ}$, $\hbox {WZZ}$, $\hbox {ZZZ}$, $\hbox {WW} \upgamma$, $\hbox {WZ} \upgamma$) processes. Simulated samples generated at NLO are also used to estimate backgrounds from associated production of single top quarks and vector bosons ($\text {tWZ}$, $\text {tZq}$, $\text {t} \upgamma$), or $\text {t} {}{\overline{\text {t}}}$ produced in association with a single boson ($\text {t} {}{\overline{\text {t}}} \hbox {W} , \text {t} {}{\overline{\text {t}}} \hbox {Z} , \text {t} {}{\overline{\text {t}}} \text {H} , \text {t} {}{\overline{\text {t}}} \upgamma$). Three separate sets of simulated events for each process are used in order to match the different data-taking conditions and algorithms used in 2016, 2017, and 2018. Most samples are generated using the MadGraph 5_amc@nlo 2.2.2 (2.4.2) program [28] at NLO for 2016 samples (2017 and 2018 samples) with at most two additional partons in the matrix element calculations. In particular, the $\text {t} {}{\overline{\text {t}}} \hbox {W}$ sample is generated with up to one additional parton, and $\text {t} {}{\overline{\text {t}}} \hbox {Z}$ and $\text {t} {}{\overline{\text {t}}} \text {H}$ with no additional partons. The $\text {t} {}{\overline{\text {t}}} \hbox {Z}$ sample, which includes $\text {t} {}{\overline{\text {t}}} \hbox {Z} /\gamma ^{*}\rightarrow \ell \ell$, is generated with a dilepton invariant mass greater than $1\,\text {GeV}$. For the $\hbox {WZ}$ sample used with 2016 conditions, as well as all $\hbox {ZZ}$ and $\text {t} {}{\overline{\text {t}}} \text {H}$ samples, the powheg box  v2 [29, 30] program is used. The MadGraph 5_amc@nlo generator at LO with up to three additional partons, scaled to NLO cross sections, is used to produce a subset of samples for some of the data taking periods: $\hbox {W} \upgamma$ (2016), $\text {t} {}{\overline{\text {t}}} \upgamma$ (2017 and 2018), $\text {tZq}$ (2018), and $\text {t} \upgamma$ (2018) [28]. Other rare backgrounds, such as $\text {t} {}{\overline{\text {t}}}$ production in association with dibosons ($\text {t} {}{\overline{\text {t}}} \hbox {WW}$, $\text {t} {}{\overline{\text {t}}} \hbox {WZ}$, $\text {t} {}{\overline{\text {t}}} \hbox {ZZ}$, $\text {t} {}{\overline{\text {t}}} \hbox {WH}$, $\text {t} {}{\overline{\text {t}}} \hbox {ZW}$, $\text {t} {}{\overline{\text {t}}} \text {HH}$) and triple top quark production ($\text {t} {}{\overline{\text {t}}} \text {t}$, $\text {t} {}{\overline{\text {t}}} \text {tW}$), are generated using LO MadGraph 5_amc@nlo without additional partons, and scaled to NLO cross sections [31]. The background from radiative top decays, with $\gamma ^{*}\rightarrow \ell \ell$, was found to be negligible in this analysis.

The top quark associated production modes for a heavy scalar (H) or pseudoscalar (A) in the mass range of [350, 650]$\,\text {GeV}$, $\text {t} {}{\overline{\text {t}}} \text {H}/\text {A}$, $\text {tqH}/\text {A}$, and $\text {tWH}/\text {A}$, with subsequent decays of $\text {H}/\text {A}$ into a pair of top quarks, are generated using LO MadGraph 5_amc@nlo, with one additional parton for all but the $\text {tqH}/\text {A}$ production mode. In the context of type-II 2HDM, these samples are scaled to LO cross sections obtained with MadGraph 5_amc@nlo model, “2HDMtII” [32, 33]. For the choice $\tan \beta = 1$ in the alignment limit [34], where $\tan \beta$ represents the ratio of vacuum expectation values of the two Higgs doublets, these cross sections reproduce those of Ref. [6], which were also used in the previous CMS result [27]. In the context of simplified models of dark matter, these samples are scaled to LO cross sections obtained with the model used in Ref. [35], which includes kinematically accessible decays of the mediator into a pair of top quarks. The processes are simulated in the narrow-width approximation, suitable for the parameter space studied here, in which the width of the mediator is 5% of its mass or less. Samples and cross sections used for constraining the modified Higgs boson propagator are generated using MadGraph 5_amc@nlo at LO, matching the prescription of Ref. [11]. Cross sections used for SM $\text {t} {}{\overline{\text {t}}} \text {t} {}{\overline{\text {t}}}$ enhanced by scalar and vector off-shell diagrams are obtained at LO from Ref. [9].

The NNPDF3.0LO (NNPDF3.0NLO) [36] parton distribution functions (PDFs) are used to generate all LO (NLO) 2016 samples, while NNPDF3.1 next-to-next-to-leading order [37] is used for 2017 and 2018 samples. Parton showering and hadronization, as well as $\hbox {W} ^{\pm }\hbox {W} ^{\pm }$ production from double-parton scattering, are modeled by the pythia  8.205 [38] program for 2016 samples and pythia  8.230 [39] for 2017 and 2018 samples, while the MLM [40] and FxFx [41] prescriptions are employed in matching additional partons from the matrix element calculations to those from parton showers for the LO and NLO samples, respectively. The underlying event modeling uses the CUETP8M1 tune [42, 43] for 2016, and CP5 [44] for 2017 and 2018 data sets, respectively. The top quark mass in the Monte Carlo programs is set to $172.5\,\text {GeV}$. The Geant4 package [45] is used to model the response of the CMS detector. Additional $\hbox {pp}$ interactions (pileup) within the same or nearby bunch crossings are also included in the simulated events.

##  The CMS detector and event reconstruction

The central feature of the CMS detector is a superconducting solenoid of 6$\,\text {m}$ internal diameter, providing a magnetic field of 3.8$\,\text {T}$. Within the solenoid volume are a silicon pixel and strip tracker, a lead tungstate crystal electromagnetic calorimeter (ECAL), and a brass and scintillator hadron calorimeter (HCAL), each composed of a barrel and two endcap sections. Forward calorimeters extend the pseudorapidity ($\eta$) coverage provided by the barrel and endcap detectors. Muons are detected in gas-ionization chambers embedded in the steel flux-return yoke outside the solenoid. A more detailed description of the CMS detector, together with a definition of the coordinate system used and the relevant variables, can be found in Ref. [46].

Events of interest are selected using a two-tiered trigger system [47]. The first level, composed of custom hardware processors, uses information from the calorimeters and muon detectors to select events at a rate of around 100$\,\text {kHz}$ within a time interval of less than 4 $\upmu$s. The second level, known as the high-level trigger, consists of a farm of processors running a version of the full event reconstruction software optimized for fast processing, and reduces the event rate to around 1$\,\text {kHz}$ before data storage.

The reconstructed vertex with the largest value of summed physics-object squared-transverse-momentum is taken to be the primary $\hbox {pp}$ interaction vertex. The physics objects are the jets, clustered using the jet finding algorithm [48, 49] with the tracks assigned to the vertex as inputs, and the associated missing transverse momentum, taken as the negative vector sum of the transverse momentum ($p_{\mathrm {T}}$) of those jets.

The particle-flow algorithm [50] aims to reconstruct and identify each individual particle in an event, with an optimized combination of information from the various elements of the CMS detector. The energy of photons is directly obtained from the ECAL measurement. The energy of electrons is determined from a combination of the electron momentum at the primary interaction vertex as determined by the tracker, the energy of the corresponding ECAL cluster, and the energy sum of all bremsstrahlung photons spatially compatible with the electron track [51]. The momentum of muons is obtained from the curvature of the corresponding track, combining information from the silicon tracker and the muon system [52]. The energy of charged hadrons is determined from a combination of their momentum measured in the tracker and the matching ECAL and HCAL energy deposits, corrected for the response function of the calorimeters to hadronic showers. The energy of neutral hadrons is obtained from the corresponding corrected ECAL and HCAL energies.

Hadronic jets are clustered from neutral PF candidates and charged PF candidates associated with the primary vertex, using the anti-$k_{\mathrm {T}}$ algorithm [48, 49] with a distance parameter of 0.4. The jet momentum is determined as the vectorial sum of all PF candidate momenta in the jet. An offset correction is applied to jet energies to take into account the contribution from pileup [53]. Jet energy corrections are derived from simulation and are improved with in situ measurements of the energy balance in dijet, multijet, $\gamma$+jet, and leptonically decaying Z+jet events [54, 55]. Additional selection criteria are applied to each jet to remove jets potentially affected by instrumental effects or reconstruction failures [56]. Jets originating from b  quarks are identified as b-tagged jets using a deep neural network algorithm, DeepCSV [57], with a working point chosen such that the efficiency to identify a b  jet is 55–70% for a jet $p_{\mathrm {T}}$ between 20 and 400$\,\text {GeV}$. The misidentification rate is approximately 1–2% for light-flavor and gluon jets and 10–15% for charm jets, in the same jet $p_{\mathrm {T}}$ range. The vector $\vec {p}_{\mathrm {T}}^{\text {miss}}$ is defined as the projection on the plane perpendicular to the beams of the negative vector sum of the momenta of all reconstructed PF candidates in an event [58]. Its magnitude, called missing transverse momentum, is referred to as $p_{\mathrm {T}} ^\text {miss}$.

## Event selection and search strategy

The identification, isolation, and impact parameter requirement with respect to the primary vertex, imposed on electrons and muons are the same as those of Ref. [27] when analyzing the 2016 data set, while for the 2017 and 2018 data sets the identification of electrons and the isolation of both electrons and muons are modified to take into account the increased pileup. For electrons, identification is based on a multivariate discriminant using shower shape and track quality variables, while muon identification is based on the quality of the geometrical matching between measurements in the tracker and the muon system. The isolation requirement, introduced in Ref. [59], is designed to distinguish the charged leptons produced in W and Z decays (“prompt leptons”) from the leptons produced in hadron decays or in conversions of photons in jets, as well as hadrons misidentified as leptons (collectively defined as “nonprompt leptons”). The requirements to minimize charge misassignment are the same as in Ref. [27]: muon tracks are required to have a small uncertainty in $p_{\mathrm {T}}$ and electron tracks are required to have the same charge as that obtained from comparing a linear projection of the pixel detector hits to the position of the calorimeter deposit. The combined efficiency to reconstruct and identify leptons is in the range of 45–80 (70–90)% for electrons (muons), increasing as a function of $p_{\mathrm {T}}$ and reaching the maximum value for $p_{\mathrm {T}} >60\,\text {GeV}$.

For the purpose of counting leptons and jets, the following requirements are applied: the number of leptons ($N_\ell$) is defined to be the multiplicity of electrons and muons with $p_{\mathrm {T}} > 20\,\text {GeV}$ and either $|\eta | < 2.5$ (electrons) or $|\eta | < 2.4$ (muons), the number of jets ($N_\text {jets}$) counts all jets with $p_{\mathrm {T}} > 40\,\text {GeV}$ and $|\eta | < 2.4$, and the number of b-tagged jets ($N_\text {b}$) counts b-tagged jets with $p_{\mathrm {T}} > 25\,\text {GeV}$ and $|\eta | < 2.4$. In order to be included in $N_\text {jets}$, $N_\text {b}$, and the $H_{\mathrm {T}}$ variable, which is defined as the scalar $p_{\mathrm {T}}$ sum of all jets in an event, jets and b-tagged jets must have an angular separation $\varDelta R > 0.4$ with respect to all selected leptons. This angular separation is defined as $\varDelta R = \sqrt{\smash [b]{(\varDelta \eta )^2+(\varDelta \phi )^2}}$, where $\varDelta \eta$ and $\varDelta \phi$ are the differences in pseudorapidity and azimuthal angle, respectively, between the directions of the lepton and the jet.

Events were recorded using either a dilepton$+$ $H_{\mathrm {T}}$ (2016) or a set of dilepton triggers (2017 and 2018). The dilepton$+$ $H_{\mathrm {T}}$ trigger requires two leptons with $p_{\mathrm {T}} > 8\,\text {GeV}$ and a minimum $H_{\mathrm {T}}$ requirement that is fully efficient with respect to the offline requirement of $300\,\text {GeV}$. The dilepton triggers require either two muons with $p_{\mathrm {T}} > 17$ and $8\,\text {GeV}$, two electrons with $p_{\mathrm {T}} > 23$ and $12\,\text {GeV}$, or an $\hbox {e} {\upmu }$ pair with $p_{\mathrm {T}} > 23\,\text {GeV}$ for the higher-$p_{\mathrm {T}}$ (leading) lepton and $p_{\mathrm {T}} > 12\ (8)\,\text {GeV}$ for the lower-$p_{\mathrm {T}}$ (trailing) electron (muon). The trigger efficiency within the detector acceptance is measured in data to be greater than $90\%$ for $\hbox {ee}$, $\hbox {e} {\upmu }$, and ${\upmu } {\upmu }$ events, and nearly $100\%$ for events with at least three leptons.

We define a baseline selection that requires $H_{\mathrm {T}} > 300\,\text {GeV}$ and $p_{\mathrm {T}} ^\text {miss} >50\,\text {GeV}$, two or more jets ($N_\text {jets} \ge 2$) and b-tagged jets ($N_\text {b} \ge 2$), a leading lepton with $p_{\mathrm {T}} > 25\,\text {GeV}$, and a trailing lepton of the same charge with $p_{\mathrm {T}} > 20\,\text {GeV}$. Events with same-sign electron pairs with an invariant mass below 12$\,\text {GeV}$ are rejected to reduce the background from production of low-mass resonances with a charge-misidentified electron. Events where a third lepton with $p_{\mathrm {T}} >7$ (5)$\,\text {GeV}$ for electrons (muons) forms an opposite-sign (OS) same-flavor pair with an invariant mass below 12$\,\text {GeV}$ or between 76 and 106$\,\text {GeV}$ are also rejected. Inverting this resonance veto, the latter events are used to populate a $\text {t} {}{\overline{\text {t}}} \hbox {Z}$ background control region (CRZ) if the invariant mass is between 76 and 106$\,\text {GeV}$ and the third lepton has $p_{\mathrm {T}} > 20\,\text {GeV}$. After this baseline selection, the signal acceptance is approximately 1.5%, including branching fractions.

Events passing the baseline selection are split into several signal and control regions, following two independent approaches. In the first analysis, similarly to Ref. [27] and referred to as “cut-based”, the variables $N_\text {jets}$, $N_\text {b}$, and $N_\ell$ are used to subdivide events into 14 mutually exclusive signal regions (SRs) and a control region (CR) enriched in $\text {t} {}{\overline{\text {t}}} \hbox {W}$ background (CRW), to complement the CRZ defined above, as detailed in Table 1. In the boosted decision tree (BDT) analysis, the CRZ is the only control region, and the remaining events are subdivided into 17 SRs by discretizing the discriminant output of a BDT trained to separate $\text {t} {}{\overline{\text {t}}} \text {t} {}{\overline{\text {t}}}$ events from the sum of the SM backgrounds.

The BDT classifier utilizes a gradient boosting algorithm to train 500 trees with a depth of 4 using simulation, and is based on the following 19 variables: $N_\text {jets}$, $N_\text {b}$, $N_\ell$, $p_{\mathrm {T}} ^\text {miss}$, $H_{\mathrm {T}}$, two alternative definitions of $N_\text {b}$ based on b  tagging working points tighter or looser than the default one, the scalar $p_{\mathrm {T}}$ sum of b-tagged jets, the $p_{\mathrm {T}}$ of the three leading leptons, of the leading jet and of the sixth, seventh, and eighth jets, the azimuthal angle between the two leading leptons, the invariant mass formed by the leading lepton and the leading jet, the charge of the leading lepton, and the highest ratio of the jet mass to the jet $p_{\mathrm {T}}$ in the event (to provide sensitivity to boosted, hadronically-decaying top quarks and W bosons). Three of the most performant input variables, $N_\text {jets}$, $N_\text {b}$, and $N_\ell$, correspond to the variables used for the cut-based analysis. Top quark tagging algorithms to identify hadronically decaying top quarks based on invariant masses of jet combinations, similarly to Ref. [23], were also tested, but did not improve the expected sensitivity. Such algorithms could only contribute in the handful of events where all the top quark decay products were found, and these events already have very small background yields. In each analysis, the observed and predicted yields in the CRs and SRs are used in a maximum likelihood fit with nuisance parameters to measure $\sigma (\hbox {pp} \rightarrow \text {t} {}{\overline{\text {t}}} \text {t} {}{\overline{\text {t}}}$), following the procedure described in Sect. 7.

**Table 1:** Definition of the 14 SRs and two CRs for the cut-based analysis

$N_\ell$	$N_\text {b}$	$N_\text {jets}$	Region
2	2	$\le$5	CRW
		6	SR1
		7	SR2
		$\ge 8$	SR3
	3	5	SR4
		6	SR5
		7	SR6
		$\ge 8$	SR7
	$\ge 4$	$\ge$5	SR8
$\ge 3$	2	5	SR9
		6	SR10
		$\ge 7$	SR11
	$\ge 3$	4	SR12
		5	SR13
		$\ge 6$	SR14
Inverted resonance veto			CRZ

## Backgrounds

In addition to the $\text {t} {}{\overline{\text {t}}} \text {t} {}{\overline{\text {t}}}$ signal, several other SM processes result in final states with same-sign dileptons or at least three leptons, and several jets and b  jets. These backgrounds primarily consist of processes where $\text {t} {}{\overline{\text {t}}}$ is produced in association with additional bosons that decay to leptons, such as $\text {t} {}{\overline{\text {t}}} \hbox {W}$, $\text {t} {}{\overline{\text {t}}} \hbox {Z}$, and $\text {t} {}{\overline{\text {t}}} \text {H}$ (mainly in the $\text {H} \rightarrow \hbox {WW}$ channel), as well as dilepton $\text {t} {}{\overline{\text {t}}}$ events with a charge-misidentified prompt-lepton and single-lepton $\text {t} {}{\overline{\text {t}}}$ events with an additional nonprompt lepton.

The prompt-lepton backgrounds, dominated by $\text {t} {}{\overline{\text {t}}} \hbox {W}$, $\text {t} {}{\overline{\text {t}}} \hbox {Z}$, and $\text {t} {}{\overline{\text {t}}} \text {H}$, are estimated using simulated events. Dedicated CRs are used to constrain the normalization for $\text {t} {}{\overline{\text {t}}} \hbox {W}$ (cut-based analysis) and $\text {t} {}{\overline{\text {t}}} \hbox {Z}$ (cut-based and BDT analyses), while for other processes described in the next paragraph, the normalization is based on the NLO cross sections referenced in Sect. 2.

Processes with minor contributions are grouped into three categories. The “$\text {t} {}{\overline{\text {t}}} \hbox {VV}$” category includes the associated production of $\text {t} {}{\overline{\text {t}}}$ with a pair of bosons ($\hbox {W}$, $\hbox {Z}$, $\text {H}$), dominated by $\text {t} {}{\overline{\text {t}}} \hbox {WW}$. The “X$\upgamma$ ” category includes processes where a photon accompanies a vector boson, a top quark, or a top-antitop quark pair. The photon undergoes a conversion, resulting in the identification of an electron in the final state. The category is dominated by $\text {t} {}{\overline{\text {t}}} \upgamma$, with smaller contributions from $\hbox {W} \upgamma$, $\hbox {Z} \upgamma$, and $\text {t} \upgamma$. Finally, the “Rare” category includes all residual processes with top quarks ($\text {tZq}$, $\text {tWZ}$, $\text {t} {}{\overline{\text {t}}} \text {t}$, and $\text {t} {}{\overline{\text {t}}} \text {tW}$) or without them ($\hbox {WZ}$, $\hbox {ZZ}$, $\hbox {W} ^{\pm }\hbox {W} ^{\pm }$ from single- and double-parton scattering, and triboson production).

Since the $\text {t} {}{\overline{\text {t}}} \hbox {W}$, $\text {t} {}{\overline{\text {t}}} \hbox {Z}$, and $\text {t} {}{\overline{\text {t}}} \text {H}$ processes constitute the largest backgrounds to $\text {t} {}{\overline{\text {t}}} \text {t} {}{\overline{\text {t}}}$ production, their simulated samples are corrected wherever possible to account for discrepancies observed between data and MC simulation. To improve the MC modeling of the additional jet multiplicity from initial-state radiation (ISR) and final-state radiation (FSR), simulated $\text {t} {}{\overline{\text {t}}} \hbox {W}$ and $\text {t} {}{\overline{\text {t}}} \hbox {Z}$ events are reweighted based on the number of ISR or FSR jets ($N_{\text {jets}}^{\mathrm {ISR/FSR}}$). The reweighting is based on a comparison of the light-flavor jet multiplicity in dilepton $\text {t} {}{\overline{\text {t}}}$ events in data and simulation, where the simulation is performed with the same generator settings as those of the $\text {t} {}{\overline{\text {t}}} \hbox {W}$ and $\text {t} {}{\overline{\text {t}}} \hbox {Z}$ samples. The method requires exactly two jets identified as originating from b  quarks in the event and assumes that all other jets are from ISR or FSR. The $N_{\text {jets}}^{\mathrm {ISR/FSR}}$ reweighting factors vary within the range of [0.77, 1.46] for $N_{\text {jets}}^{\mathrm {ISR/FSR}}$ between 1 and 4. This correction is not applied to $\text {t} {}{\overline{\text {t}}} \text {H}$ ($\text {H} \rightarrow \hbox {WW}$) events, which already have additional jets from the decay of the additional $\hbox {W}$ bosons. In addition to the ISR or FSR correction, the $\text {t} {}{\overline{\text {t}}} \hbox {W}$, $\text {t} {}{\overline{\text {t}}} \hbox {Z}$, and $\text {t} {}{\overline{\text {t}}} \text {H}$ simulation is corrected to improve the modeling of the flavor of additional jets, based on the measured ratio of the $\text {t} {}{\overline{\text {t}}} \,\hbox {b}{\bar{\text {b}}}$ and $\text {t} {}{\overline{\text {t}}} \,\text {jj}$ cross sections, $1.7\pm 0.6$ , reported in Ref. [60], where j represents a generic jet. This correction results in a 70% increase of events produced in association with a pair of additional b  jets. Other topologies, such as those including c quarks, are negligible by comparison, and no dedicated correction is performed.

The nonprompt lepton backgrounds are estimated using the “tight-to-loose” ratio method [59]. The tight identification (for electrons) and isolation (for both electrons and muons) requirements of the SRs are relaxed to define a loose lepton selection, enriched in nonprompt leptons. The efficiency, $\epsilon _{\mathrm {TL}}$, for nonprompt leptons that satisfy the loose selection to also satisfy the tight selection is measured in a control sample of single-lepton events, as a function of lepton flavor, $p_{\mathrm {T}}$, and $|\eta |$, after subtracting the prompt-lepton contamination based on simulation. The loose selection is chosen to ensure that $\epsilon _{\mathrm {TL}}$ remains stable across the main categories of nonprompt leptons specified in Sect. 4, allowing the same $\epsilon _{\mathrm {TL}}$ to be applied to samples with different nonprompt lepton composition. For leptons failing the tight selection, the $p_{\mathrm {T}}$ variable is redefined as the sum of the lepton $p_{\mathrm {T}}$ and the energy in the isolation cone exceeding the isolation threshold value. This parametrization accounts for the momentum spectrum of the parent parton (the parton that produced the nonprompt lepton), allowing the same $\epsilon _{\mathrm {TL}}$ to be applied to samples with different parent parton momenta with reduced bias. To estimate the number of nonprompt leptons in each SR, a dedicated set of application regions is defined, requiring at least one lepton to fail the tight selection while satisfying the loose one (loose-not-tight). Events in these regions are then weighted by a factor of $\epsilon _{\mathrm {TL}} / (1-\epsilon _{\mathrm {TL}})$ for each loose-not-tight lepton. To avoid double counting the contribution of events with multiple nonprompt leptons, events with two loose-not-tight leptons are subtracted, and the resulting total weight is used as a prediction of the nonprompt lepton yield.

The background resulting from charge-misidentified leptons is estimated using the charge-misidentification probability measured in simulation as a function of electron $p_{\mathrm {T}}$ and $|\eta |$. This probability ranges between $10^{-5}$ and $10^{-3}$ for electrons and is at least an order of magnitude smaller for muons. Charge-misidentified muons are therefore considered negligible, while for electrons this probability is applied to a CR of OS dilepton events defined for each same-sign dilepton SR. A single correction factor, inclusive in $p_{\mathrm {T}}$ and $|\eta |$, is applied to the resulting estimate to account for differences between data and simulation in this probability. A correction factor, derived from a control sample enriched in $\hbox {Z} \rightarrow {{\hbox {e}}^{+}} {{\hbox {e}}^{-}}$ events with one electron or positron having a misidentified charge, is very close to unity for the 2016 simulation, while it is approximately 1.4 for the 2017 and 2018 simulation. Even with the larger correction factors, the charge-misidentification probability is smaller in 2017 and 2018 than in 2016, due to the upgraded pixel detector [61].

## Uncertainties

Several sources of experimental and theoretical uncertainty related to signal and background processes are considered in this analysis. They are summarized, along with their estimated correlation treatment across the 2016, 2017, and 2018 data sets, in Table 2. Most sources of uncertainties affect simulated samples, while the backgrounds obtained using control samples in data (charge-misidentified and nonprompt leptons) have individual uncertainties described at the end of this section.

The uncertainties in the integrated luminosity are 2.5, 2.3, and 2.5% for the 2016, 2017, and 2018 data collection periods, respectively [62–64]. Simulated events are reweighted to match the distribution of the number of pileup collisions per event in data. This distribution is derived from the instantaneous luminosity and the inelastic cross section [65], and uncertainties in the latter are propagated to the final yields, resulting in yield variations of at most 5%.

**Table 2:** Summary of the sources of uncertainty, their values, and their impact, defined as the relative change of the measurement of $\sigma (\text {t} {}{\overline{\text {t}}} \text {t} {}{\overline{\text {t}}} )$ induced by one-standard-deviation variations corresponding to each uncertainty source considered separately. The first group lists experimental and theoretical uncertainties in simulated signal and background processes. The second group lists normalization uncertainties in the estimated backgrounds. Uncertainties marked (not marked) with a $\dagger$ in the first column are treated as fully correlated (fully uncorrelated) across the 3 years of data taking

		Impact on
Source	Uncertainty (%)	$\sigma (\text {t} {}{\overline{\text {t}}} \text {t} {}{\overline{\text {t}}} )$ (%)
Integrated luminosity	2.3–2.5	2
Pileup	0–5	1
Trigger efficiency	2–7	2
Lepton selection	2–10	2
Jet energy scale	1–15	9
Jet energy resolution	1–10	6
$\text {b}$ tagging	1–15	6
Size of simulated sample	1–25	<1
Scale and PDF variations $\dagger$	10–15	2
ISR/FSR (signal) $\dagger$	5–15	2
$\text {t} {}{\overline{\text {t}}} \text {H}$ (normalization) $\dagger$	25	5
Rare, X$\upgamma$, $\text {t} {}{\overline{\text {t}}} \hbox {VV}$ (norm.) $\dagger$	11–20	<1
$\text {t} {}{\overline{\text {t}}} \hbox {Z}$, $\text {t} {}{\overline{\text {t}}} \hbox {W}$ (norm.) $\dagger$	40	3–4
Charge misidentification $\dagger$	20	<1
Nonprompt leptons ${}\dagger$	30–60	3
$N_{\text {jets}}^{\mathrm {ISR/FSR}}$	1–30	2
$\sigma ({\text {t} {}{\overline{\text {t}}} \,\hbox {b}{\bar{\text {b}}}})/\sigma ({\text {t} {}{\overline{\text {t}}} \,\mathrm {jj}})$ ${}\dagger$	35	11

The efficiency of the trigger requirements is measured in an independent data sample selected using single-lepton triggers, with an uncertainty of 2%. The lepton reconstruction and identification efficiency is measured using a data sample enriched in $\hbox {Z} \rightarrow \ell \ell$ events [51, 52], with uncertainties of up to 5 (3)% per electron (muon). The tagging efficiencies for b  jets and light-flavor jets are measured in dedicated data samples [57], and their uncertainties result in variations between 1 and 15% of the signal region yields. In all cases, simulated events are reweighted to match the efficiencies measured in data. The uncertainty associated with jet energy corrections results in yield variations of 1–15% across SRs. Uncertainties in the jet energy resolution result in 1–10% variations [54].

As discussed in Sect. 5, we correct the distribution of the number of additional jets in $\text {t} {}{\overline{\text {t}}} \hbox {W}$ and $\text {t} {}{\overline{\text {t}}} \hbox {Z}$ samples, with reweighting factors varying within the range of [0.77, 1.46]. We take one half of the differences from unity as the systematic uncertainties in these factors, since they are measured in a $\text {t} {}{\overline{\text {t}}}$ sample, but are applied to different processes. These uncertainties result in yield variations up to 8% across SRs. Similarly, events with additional b  quarks in $\text {t} {}{\overline{\text {t}}} \hbox {W}$, $\text {t} {}{\overline{\text {t}}} \hbox {Z}$, and $\text {t} {}{\overline{\text {t}}} \text {H}$ are scaled by a factor of $1.7\pm 0.6$, based on the CMS measurement of the ratio of cross sections $\sigma ({\text {t} {}{\overline{\text {t}}} \,\hbox {b}{\bar{\text {b}}}})/\sigma ({\text {t} {}{\overline{\text {t}}} \,\mathrm {jj}})$ [60]. The resulting uncertainty in the yields for SRs with $N_\text {b} \ge 4$, where the effect is dominant, is up to 15%.

For background processes, uncertainties in the normalization (number of events passing the baseline selection) and shape (distribution of events across SRs) are considered, while for signal processes, the normalization is unconstrained, and instead, we consider the uncertainty in the acceptance (fraction of events passing the baseline selection) and shape. For each of the Rare, X$\upgamma$, and $\text {t} {}{\overline{\text {t}}} \hbox {VV}$ categories, normalization uncertainties are taken from the largest theoretical cross section uncertainty in any constituent physics process, resulting in uncertainties of 20%, 11%, and 11%, respectively. For the $\text {t} {}{\overline{\text {t}}} \hbox {W}$ and $\text {t} {}{\overline{\text {t}}} \hbox {Z}$ processes, we set an initial normalization uncertainty of 40%, but then allow the maximum-likelihood fit to constrain these backgrounds further using control samples in data. For $\text {t} {}{\overline{\text {t}}} \text {H}$, we assign a 25% normalization uncertainty to reflect the signal strength, which is the ratio between the measured cross section of $\text {t} {}{\overline{\text {t}}} \text {H}$ and its SM expectation, of $1.26^{+0.31}_{-0.26}$ measured by CMS [66].

The shape uncertainty resulting from variations of the renormalization and factorization scales in the event generators is smaller than 15% for backgrounds, and 10% for the $\text {t} {}{\overline{\text {t}}} \text {t} {}{\overline{\text {t}}}$ and 2HDM signals, while the effect of the PDFs is only 1%. For the $\text {t} {}{\overline{\text {t}}} \text {t} {}{\overline{\text {t}}}$ and 2HDM signals, the uncertainty in the acceptance from variations of the scales is 2%. The uncertainty in the scales that determine ISR and FSR, derived from $\text {t} {}{\overline{\text {t}}} \text {t} {}{\overline{\text {t}}}$ samples, results in up to 6 and 10% uncertainties in signal acceptance and shape, respectively. When considering $\text {t} {}{\overline{\text {t}}} \text {t} {}{\overline{\text {t}}}$ as a background in BSM interpretations, a cross section uncertainty of 20% (based on the prediction of $12.0^{+2.2}_{-2.5}\,\text {fb}$ [1]) is additionally applied to the $\text {t} {}{\overline{\text {t}}} \text {t} {}{\overline{\text {t}}}$ process.

The charge-misidentified and nonprompt-lepton backgrounds are assigned an uncertainty of 20 and 30%, respectively, where the latter is increased to 60% for nonprompt electrons with $p_{\mathrm {T}} > 50\,\text {GeV}$. For the charge-misidentified lepton background, the uncertainty is based on the agreement observed between the prediction and data as a function of kinematic distributions, in a data sample enriched in $\hbox {Z} \rightarrow {{\hbox {e}}^{+}} {{\hbox {e}}^{-}}$ events with one electron or positron having a misidentified charge. For the nonprompt-lepton background, the uncertainty is based on the agreement observed in simulation closure tests of the “tight-to-loose” method using multijet, $\text {t} {}{\overline{\text {t}}}$, and W $+\!$ jets samples. The contamination of prompt leptons, which is subtracted based on simulation, is below 1% in the application region, but it can be significant in the control sample where $\epsilon _{\mathrm {TL}}$ is measured, resulting in an uncertainty up to 50% in $\epsilon _{\mathrm {TL}}$. The statistical uncertainty in the estimate based on control samples in data is taken into account for both backgrounds. It is negligible for the charge-misidentified lepton background, while for the nonprompt-lepton background it can be comparable or larger than the systematic uncertainty.

Experimental uncertainties in normalization and shape are treated as fully correlated among the SRs for all signal and background processes. Two choices of correlation across years (uncorrelated or fully correlated) were tested for each experimental uncertainty, and their impact on the measurement of $\sigma (\text {t} {}{\overline{\text {t}}} \text {t} {}{\overline{\text {t}}} )$ was found to be smaller than 1%. For simplicity, these uncertainties are then treated as uncorrelated. Systematic uncertainties in the background estimates based on control samples in data and theoretical uncertainties in the normalization of each background process are treated as uncorrelated between processes but fully correlated among the SRs and across the 3 years. Scale and PDF uncertainties, as well as uncertainties in the number of additional b  quarks, are correlated between processes, signal regions, and years. Statistical uncertainties due to the finite number of simulated events or control region events are considered uncorrelated.

## Results

**Fig. 2:**
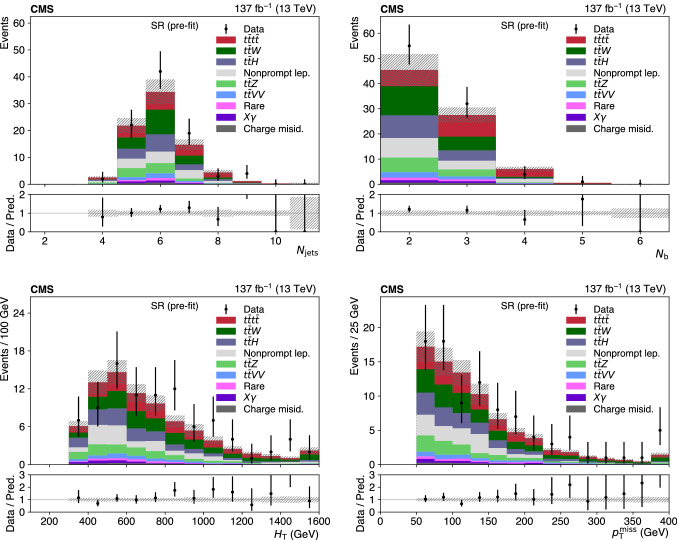
Distributions of $N_\text {jets}$ (upper left), $N_\text {b}$ (upper right), $H_{\mathrm {T}}$ (lower left), and $p_{\mathrm {T}} ^\text {miss}$ (lower right) in the summed SRs (1–14), before fitting to data, where the last bins include the overflows. The hatched areas represent the total uncertainties in the SM signal and background\,predictions. The $\text {t} {}{\overline{\text {t}}} \text {t} {}{\overline{\text {t}}}$ signal assumes the SM cross section from Ref. [1]. The lower panels show the ratios of the observed event yield to the total prediction of signal plus background

**Fig. 3:**
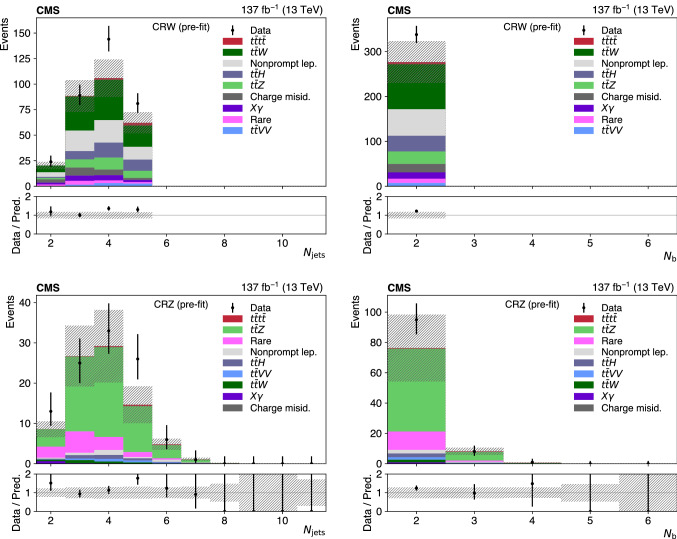
Distributions of $N_\text {jets}$ (left) and $N_\text {b}$ (right) in the $\text {t} {}{\overline{\text {t}}} \hbox {W}$ (upper) and $\text {t} {}{\overline{\text {t}}} \hbox {Z}$ (lower) CRs, before fitting to data. The hatched areas represent the uncertainties in the SM signal and background predictions. The $\text {t} {}{\overline{\text {t}}} \text {t} {}{\overline{\text {t}}}$ signal assumes the SM cross section from Ref. [1]. The lower panels show the ratios of the observed event yield to the total prediction of signal plus background

Distributions of the main kinematic variables ($N_\text {jets}$, $N_\text {b}$, $H_{\mathrm {T}}$, and $p_{\mathrm {T}} ^\text {miss}$) for events in the baseline region, as defined in Sect. 4, are shown in Fig. 2 and compared to the SM background predictions. The $N_\text {jets}$ and $N_\text {b}$ distributions for the CRW and CRZ are shown in Fig. 3. The expected SM $\text {t} {}{\overline{\text {t}}} \text {t} {}{\overline{\text {t}}}$ signal, normalized to its predicted cross section, is shown in both figures. The SM predictions are statistically consistent with the observations.

A binned likelihood is constructed using the yields from the signal regions, the CRZ, as well as the CRW for the cut-based analysis only, incorporating the experimental and theoretical uncertainties described in Sect. 6 as “nuisance” parameters. The measured cross section for $\text {t} {}{\overline{\text {t}}} \text {t} {}{\overline{\text {t}}}$ and the significance of the observation relative to the background-only hypothesis are obtained from a profile maximum-likelihood fit, in which the parameter of interest is $\sigma (\hbox {pp} \rightarrow \text {t} {}{\overline{\text {t}}} \text {t} {}{\overline{\text {t}}}$) and all nuisance parameters are profiled, following the procedures described in Refs. [22, 67]. In addition, an upper limit at 95% confidence level ($\text {CL}$) is set on $\sigma (\hbox {pp} \rightarrow \text {t} {}{\overline{\text {t}}} \text {t} {}{\overline{\text {t}}}$) using the modified frequentist $\text {CL}_\text {s}$ criterion [68, 69], with the profile likelihood ratio test statistic and asymptotic approximation [70]. We verified the consistency between the asymptotic and fully toy-based methods. Alternatively, by considering the SM, including the $\text {t} {}{\overline{\text {t}}} \text {t} {}{\overline{\text {t}}}$ process with the SM cross section and uncertainty [1], as the null hypothesis, the fit provides cross section upper limits on BSM processes with new scalar and pseudoscalar particles, as discussed in Sect. 8.

**Fig. 4:**
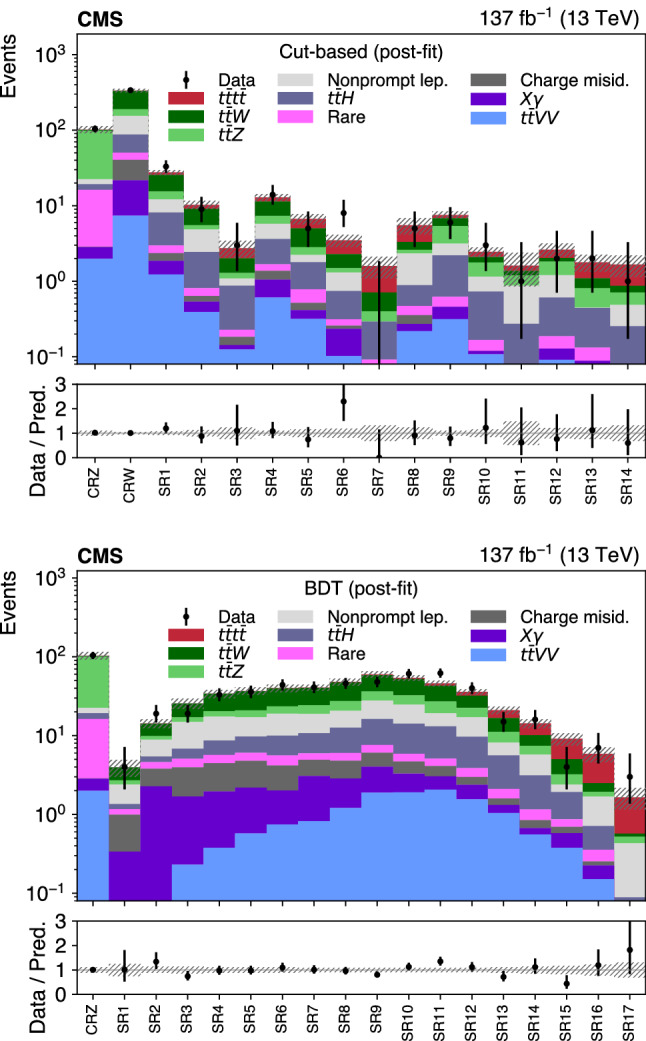
Observed yields in the control and signal regions for the cut-based (upper) and BDT (lower) analyses, compared to the post-fit predictions for signal and background processes. The hatched areas represent the total post-fit uncertainties in the signal and background predictions. The lower panels show the ratios of the observed event yield to the total prediction of signal plus background

The values and uncertainties of most nuisance parameters are unchanged by the fit, but the ones significantly affected include those corresponding to the $\text {t} {}{\overline{\text {t}}} \hbox {W}$ and $\text {t} {}{\overline{\text {t}}} \hbox {Z}$ normalizations, which are both scaled by $1.3\pm 0.2$ by the fit, in agreement with the ATLAS and CMS measurements of these processes [71–73]. The predicted yields after the maximum-likelihood fit (post-fit) are compared to data in Fig. 4 for the cut-based (upper) and BDT (lower) analyses, where the fitted $\text {t} {}{\overline{\text {t}}} \text {t} {}{\overline{\text {t}}}$ signal contribution is added to the background predictions. The corresponding yields are shown in Tables 3 and 4 for the cut-based and BDT analysis, respectively.

The $\text {t} {}{\overline{\text {t}}} \text {t} {}{\overline{\text {t}}}$ cross section and the 68% $\text {CL}$ interval is measured to be $9.4^{+6.2}_{-5.6}\,\text {fb}$ in the cut-based analysis, and $12.6^{+5.8}_{-5.2}\,\text {fb}$ in the BDT analysis. Relative to the background-only hypothesis, the observed and expected significances are 1.7 and 2.5 standard deviations, respectively, for the cut-based analysis, and 2.6 and 2.7 standard deviations for the BDT analysis. The observed 95% $\text {CL}$ upper limits on the cross section are $20.0\,\text {fb}$ in the cut-based and $22.5\,\text {fb}$ in the BDT analyses. The corresponding expected upper limits on the $\text {t} {}{\overline{\text {t}}} \text {t} {}{\overline{\text {t}}}$ cross section, assuming no SM $\text {t} {}{\overline{\text {t}}} \text {t} {}{\overline{\text {t}}}$ contribution to the data, are $9.4^{+4.3}_{-2.9}\,\text {fb}$ (cut-based) and $8.5^{+3.9}_{-2.6}\,\text {fb}$ (BDT), a significant improvement relative to the value of $20.8^{+11.2}_{-6.9}\,\text {fb}$ of Ref. [27]. The BDT and cut-based observed results were found to be statistically compatible by using correlated toy pseudo-data sets. We consider the BDT analysis as the primary result of this paper, as it provides a higher expected measurement precision, and use the results from it for further interpretations in the following section.

**Table 3:** The post-fit predicted background, $\text {t} {}{\overline{\text {t}}} \text {t} {}{\overline{\text {t}}}$ signal, and total yields with their total uncertainties and the observed number of events in the control and signal regions in data for the cut-based analysis

	SM background	$\text {t} {}{\overline{\text {t}}} \text {t} {}{\overline{\text {t}}}$	Total	Observed
CRZ	$$101\pm 10\ \ $$	$0.83\pm 0.49$	$$102\pm 10\ \ $$	104
CRW	$$331\pm 19\ \ $$	$3.9\pm 2.3$	$$335\pm 18\ \ $$	338
SR1	$$25.6\pm 2.1\ \ $$	$2.0\pm 1.2$	$$27.6\pm 2.1\ \ $$	33
SR2	$9.1\pm 1.3$	$1.13\pm 0.65$	$$10.3\pm 1.3\ \ $$	9
SR3	$2.01\pm 0.58$	$0.73\pm 0.42$	$2.74\pm 0.67$	3
SR4	$$11.3\pm 1.3\ \ $$	$1.58\pm 0.90$	$$12.9\pm 1.3\ \ $$	14
SR5	$5.03\pm 0.77$	$1.68\pm 0.95$	$6.7\pm 1.1$	5
SR6	$2.29\pm 0.40$	$1.20\pm 0.67$	$3.48\pm 0.66$	8
SR7	$0.71\pm 0.20$	$0.88\pm 0.48$	$1.59\pm 0.49$	0
SR8	$3.31\pm 0.95$	$2.2\pm 1.3$	$5.5\pm 1.3$	5
SR9	$6.84\pm 0.80$	$0.71\pm 0.39$	$7.55\pm 0.80$	6
SR10	$2.10\pm 0.31$	$0.35\pm 0.22$	$2.45\pm 0.35$	3
SR11	$1.38\pm 0.75$	$0.23\pm 0.14$	$1.61\pm 0.75$	1
SR12	$2.03\pm 0.48$	$0.59\pm 0.34$	$2.62\pm 0.54$	2
SR13	$1.09\pm 0.28$	$0.69\pm 0.39$	$1.78\pm 0.44$	2
SR14	$0.87\pm 0.30$	$0.80\pm 0.45$	$1.67\pm 0.52$	1

**Table 4:** The post-fit predicted background and $\text {t} {}{\overline{\text {t}}} \text {t} {}{\overline{\text {t}}}$ signal, and total yields with their total uncertainties and the observed number of events in the control and signal regions in data for the BDT analysis

	SM background	$\text {t} {}{\overline{\text {t}}} \text {t} {}{\overline{\text {t}}}$	Total	Observed
CRZ	$$102\pm 12\ \ $$	$1.11\pm 0.43$	$$103\pm 12\ \ $$	104
SR1	$3.95\pm 0.96$	$<0.01$	$3.96\pm 0.96$	4
SR2	$$14.2\pm 1.8\ \ $$	$0.01\pm 0.01$	$$14.2\pm 1.8\ \ $$	19
SR3	$$25.5\pm 3.5\ \ $$	$0.04\pm 0.03$	$$25.6\pm 3.5\ \ $$	19
SR4	$$34.0\pm 4.0\ \ $$	$0.08\pm 0.05$	$$34.0\pm 4.0\ \ $$	33
SR5	$$36.7\pm 4.0\ \ $$	$0.15\pm 0.07$	$$36.8\pm 4.0\ \ $$	36
SR6	$$39.8\pm 4.2\ \ $$	$0.23\pm 0.12$	$$40.0\pm 4.2\ \ $$	44
SR7	$$40.3\pm 3.7\ \ $$	$0.31\pm 0.16$	$$40.6\pm 3.8\ \ $$	41
SR8	$$47.3\pm 4.3\ \ $$	$0.72\pm 0.28$	$$48.0\pm 4.3\ \ $$	46
SR9	$$58.5\pm 5.2\ \ $$	$1.18\pm 0.46$	$$59.7\pm 5.2\ \ $$	48
SR10	$$52.1\pm 4.3\ \ $$	$1.91\pm 0.74$	$$54.1\pm 4.2\ \ $$	61
SR11	$$43.0\pm 3.5\ \ $$	$3.0\pm 1.2$	$$46.0\pm 3.5\ \ $$	62
SR12	$$32.1\pm 3.0\ \ $$	$3.7\pm 1.4$	$$35.8\pm 2.9\ \ $$	40
SR13	$$16.7\pm 1.6\ \ $$	$4.3\pm 1.6$	$$21.0\pm 2.0\ \ $$	15
SR14	$$10.1\pm 1.2\ \ $$	$4.2\pm 1.6$	$$14.3\pm 1.8\ \ $$	16
SR15	$5.03\pm 0.77$	$4.1\pm 1.5$	$9.1\pm 1.6$	4
SR16	$2.49\pm 0.61$	$3.4\pm 1.3$	$5.9\pm 1.3$	7
SR17	$0.57\pm 0.36$	$1.08\pm 0.42$	$1.65\pm 0.50$	3

## Interpretations

This analysis is used to constrain SM parameters, as well as production of BSM particles and operators that can affect the $\text {t} {}{\overline{\text {t}}} \text {t} {}{\overline{\text {t}}}$ production rate. The existence of $\text {t} {}{\overline{\text {t}}} \text {t} {}{\overline{\text {t}}}$ Feynman diagrams with virtual Higgs bosons allows interpreting the upper limit on $\sigma (\hbox {pp} \rightarrow \text {t} {}{\overline{\text {t}}} \text {t} {}{\overline{\text {t}}}$) as a constraint on the Yukawa coupling, $y_{\text {t}}$, between the top quark and the Higgs boson [2, 3]. Similarly, the measurement can be interpreted as a constraint on the Higgs boson oblique parameter $\hat{H}$, defined as the Wilson coefficient of the dimension-six BSM operator modifying the Higgs boson propagator [11]. More generically, Feynman diagrams where the virtual Higgs boson is replaced by a virtual BSM scalar ($\phi$) or vector (${\hbox {Z}}^{\prime }$) particle with mass smaller than twice the top quark mass ($m < 2m_\text {t}$), are used to interpret the result as a constraint on the couplings of such new particles [9]. In addition, new particles with $m > 2m_\text {t}$, such as a heavy scalar (H) or pseudoscalar (A), can be produced on-shell in association with top quarks. They can subsequently decay into top quark pairs, generating final states with three or four top quarks. Constraints on the production of such heavy particles can be interpreted in terms of 2HDM parameters [4–6], or in the framework of simplified models of dark matter [7, 8].

**Fig. 5:**
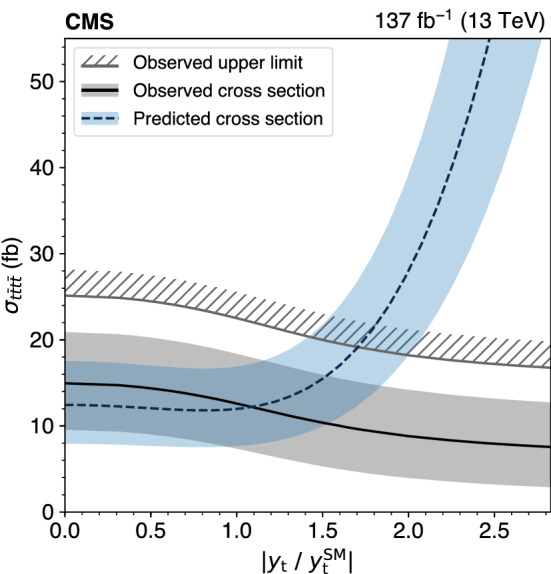
The observed $\sigma (\hbox {pp} \rightarrow \text {t} {}{\overline{\text {t}}} \text {t} {}{\overline{\text {t}}}$) (solid line) and 95% $\text {CL}$ upper limit (hatched line) are shown as a function of $|y_{\text {t}}/y_{\text {t}}^{\mathrm {SM}} |$. The predicted value (dashed line) [2], calculated at LO and scaled to the calculation from Ref. [1], is also plotted. The shaded band around the measured value gives the total uncertainty, while the shaded band around the predicted curve shows the theoretical uncertainty associated with the renormalization and factorization scales

When using our $\text {t} {}{\overline{\text {t}}} \text {t} {}{\overline{\text {t}}}$ to determine a constraint on $y_{\text {t}}$, we verified using a LO simulation that the signal acceptance is not affected by the relative contribution of the virtual Higgs boson Feynman diagrams. We take into account the dependence of the backgrounds on $y_{\text {t}}$ by scaling the $\text {t} {}{\overline{\text {t}}} \text {H}$ cross section by $|y_{\text {t}}/y_{\text {t}}^{\mathrm {SM}} |^2$ prior to the fit, where $y_{\text {t}}^{\mathrm {SM}}$ represents the SM value of the top quark Yukawa coupling. As a result of the $\text {t} {}{\overline{\text {t}}} \text {H}$ background rescaling, the measured $\sigma (\hbox {pp} \rightarrow \text {t} {}{\overline{\text {t}}} \text {t} {}{\overline{\text {t}}}$) depends on $|y_{\text {t}}/y_{\text {t}}^{\mathrm {SM}} |$, as shown in Fig. 5. The measurement is compared to the theoretical prediction obtained from the LO calculation of Ref. [2], scaled to the $12.0^{+2.2}_{-2.5}\,\text {fb}$ cross section obtained in Ref. [1], and including the uncertainty associated with doubling and halving the renormalization and factorization scales. Comparing the observed limit on $\sigma (\hbox {pp} \rightarrow \text {t} {}{\overline{\text {t}}} \text {t} {}{\overline{\text {t}}}$) with the central, upper, and lower values of its theoretical prediction, we obtain 95% $\text {CL}$ limits of $|y_{\text {t}}/y_{\text {t}}^{\mathrm {SM}} | < 1.7$, 1.4, and 2.0, respectively, an improvement over the previous CMS result [27]. Alternatively, assuming that the on-shell Yukawa coupling is equal to that of the SM, we do not rescale the $\text {t} {}{\overline{\text {t}}} \text {H}$ background with respect to its SM prediction, and obtain corresponding limits on the off-shell Yukawa coupling of $|y_{\text {t}}/y_{\text {t}}^{\mathrm {SM}} | < 1.8$, 1.5, and 2.1. Since $y_{\text {t}}$ affects the Higgs boson production cross section in both the gluon fusion and $\text {t} {}{\overline{\text {t}}} \text {H}$ modes, constraints on $y_{\text {t}}$ can also be obtained from a combination of Higgs boson measurements [74]. However, these constraints require assumptions about the total width of the Higgs boson, while the $\text {t} {}{\overline{\text {t}}} \text {t} {}{\overline{\text {t}}}$-based limit does not. For the $\hat{H}$ interpretation, the BDT analysis is repeated using simulated samples of $\text {t} {}{\overline{\text {t}}} \text {t} {}{\overline{\text {t}}}$ signal events with different values of $\hat{H}$ to account for small acceptance and kinematic differences, as described in Sect. 2. We rescale the $\text {t} {}{\overline{\text {t}}} \text {H}$ cross section by $(1-\hat{H})^2$ to account for its $\hat{H}$ dependency [11]. This results in the 95% $\text {CL}$ upper limit of $\hat{H} < 0.12$. For reference, the authors of Ref. [11] used recent LHC on-shell Higgs boson measurements to set a constraint of $\hat{H} < 0.16$ at 95% $\text {CL}$.

**Fig. 6:**
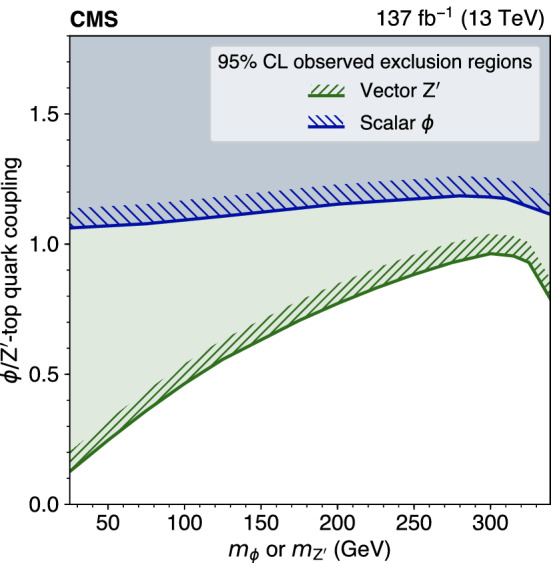
The 95% $\text {CL}$ exclusion regions in the plane of the $\phi /{\hbox {Z}}^{\prime }$-top quark coupling versus $m_{\phi }$ or $m_{\mathrm{Z}^{\prime }}$. The excluded regions are above the hatched lines

To study the off-shell effect of new particles with $m < 2m_\text {t}$, we first consider neutral scalar ($\phi$) and neutral vector (${\hbox {Z}}^{\prime }$) particles that couple to top quarks. Such particles are at present only weakly constrained, while they can give significant contributions to the $\text {t} {}{\overline{\text {t}}} \text {t} {}{\overline{\text {t}}}$ cross section [9]. Having verified in LO simulation that these new particles affect the signal acceptance by less than 10%, we recalculate the $\sigma (\hbox {pp} \rightarrow \text {t} {}{\overline{\text {t}}} \text {t} {}{\overline{\text {t}}}$) upper limit of the BDT analysis including an additional 10% uncertainty in the acceptance, and obtain the 95% $\text {CL}$ upper limit of 23.0$\,\text {fb}$ on the total $\text {t} {}{\overline{\text {t}}} \text {t} {}{\overline{\text {t}}}$ cross section, slightly weaker than the 22.5$\,\text {fb}$ limit obtained in Sect. 7. Comparing this upper limit to the predicted cross section in models where $\text {t} {}{\overline{\text {t}}} \text {t} {}{\overline{\text {t}}}$ production includes a $\phi$ or a ${\hbox {Z}}^{\prime }$ in addition to SM contributions and associated interference, we set limits on the masses and couplings of these new particles, shown in Fig. 6. These limits exclude couplings larger than 1.2 for $m_{\phi }$ in the 25–340$\,\text {GeV}$ range and larger than 0.1 (0.9) for $m_\mathrm{Z^{\prime }} = 25$ (300)$\,\text {GeV}$.

**Fig. 7:**
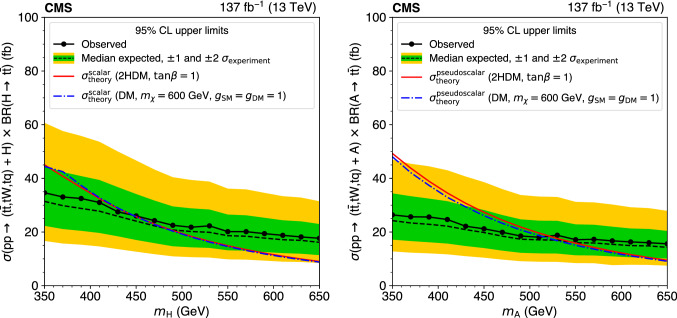
The observed (points) and expected (dashed line) 95% $\text {CL}$ upper limits on the cross section times branching fraction to $\text {t} {}{\overline{\text {t}}}$ for the production of a new heavy scalar H (left) and pseudoscalar A (right), as a function of mass. The inner and outer bands around the expected limits indicate the regions containing 68 and 95%, respectively, of the distribution of limits under the background-only hypothesis. Theoretical values are shown for Type-II 2HDM in the alignment limit (solid line) and simplified dark matter (dot-dashed line) models

**Fig. 8:**
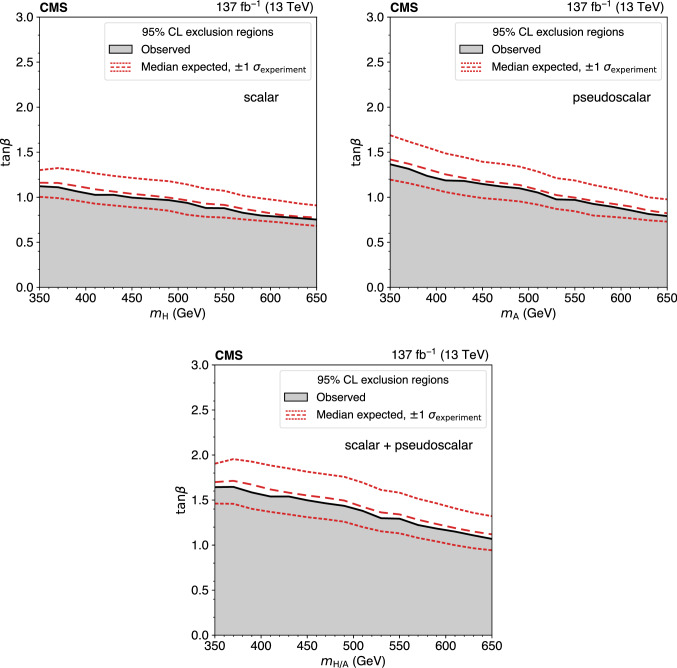
The observed (solid curve) and expected (long-dashed curve) 95% $\text {CL}$ exclusion regions in the $\tan \beta$ versus mass plane for Type-II 2HDM models in the alignment limit for a new scalar H (upper left), pseudoscalar A (upper right), and both (lower) particles. The short-dashed curves around the expected limits indicate the region containing 68% of the distribution of limits expected under the background-only hypothesis. The excluded regions are below the curves

**Fig. 9:**
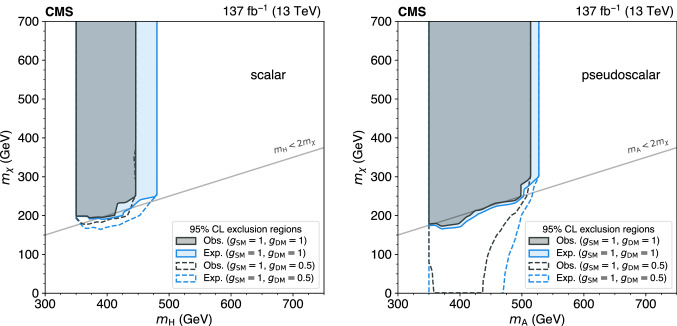
Exclusion regions at 95% $\text {CL}$ in the plane of $m_\chi$ vs. $m_{\text {H}}$ (left) or $m_{\text {A}}$ (right). The outer lighter and inner darker solid curves show the expected and observed limits, respectively, assuming $g_\mathrm {SM} = g_\mathrm {DM} = 1$. The excluded regions, shaded, are above the limit curves. The dashed lines show the limits assuming a weaker coupling between $\text {H}/\text {A}$ and $\chi$, $g_\mathrm {DM} = 0.5$

We consider on-shell effects from new scalar and pseudoscalar particles with $m > 2m_\text {t}$. At such masses, the production rate of these particles in association with a single top quark ($\text {tq} \text {H}/\text {A}$, $\text {tW} \text {H}/\text {A}$) becomes significant, so we include these processes in addition to $\text {t} \overline{\text {t}}\text {H}/\text {A}$. As pointed out in Ref. [6], these processes do not suffer significant interference with the SM $\text {t} {}{\overline{\text {t}}} \text {t} {}{\overline{\text {t}}}$ process. To obtain upper limits on the sum of these processes followed by the decay $\text {H}/\text {A} \rightarrow \text {t} {}{\overline{\text {t}}}$, we use the BDT analysis and treat the SM $\text {t} {}{\overline{\text {t}}} \text {t} {}{\overline{\text {t}}}$ process as a background. Figure 7 shows the excluded cross section as a function of the mass of the scalar (left) and pseudoscalar (right). Comparing these limits with the Type-II 2HDM cross sections with $\tan \beta = 1$ in the alignment limit, we exclude scalar (pseudoscalar) masses up to 470 (550)$\,\text {GeV}$, improving by more than 100$\,\text {GeV}$ with respect to the previous CMS limits [26]. Alternatively, we consider the simplified model of dark matter defined in Ref. [35], which includes a Dirac fermion dark matter candidate, $\chi$, in addition to $\text {H}/\text {A}$, and where the couplings of $\text {H}/\text {A}$ to SM fermions and $\chi$ are determined by parameters $g_\mathrm {SM}$ and $g_\mathrm {DM}$, respectively. In this model, exclusions similar to those from 2HDM are reached by assuming $g_\mathrm {SM} = 1$ and $g_\mathrm {DM} = 1$, and taking $m_{\text {H}/\text {A}} < 2 m_\chi$. Relaxing the 2HDM assumption of $\tan \beta = 1$, Fig. 8 shows the 2HDM limit as a function of $\text {H}/\text {A}$ mass and $\tan \beta$, considering one new particle at a time and also including a scenario with $m_\text {H} = m_\text {A}$ inspired by a special case of Type-II 2HDM, the hMSSM [75]. Values of $\tan \beta$ up to 0.8–1.6 are excluded, depending on the assumptions made. These exclusions are comparable to those of a recent CMS search for the resonant production of $\text {H}/\text {A}$ in the $\hbox {p} \rightarrow \text {H}/\text {A} \rightarrow \text {t} {}{\overline{\text {t}}}$ channel [76]. Relaxing the $m_{\text {H}/\text {A}} < 2 m_\chi$ assumption in the dark matter model, Fig. 9 shows the limit in this model as a function of the masses of both $\text {H}/\text {A}$ and $\chi$, for $g_\mathrm {DM} = 1$ and for two different assumptions of $g_\mathrm {SM}$. Large sections of the phase space of simplified dark matter models are excluded, and the reach of this analysis is complementary to that of analyses considering decays of $\text {H}/\text {A}$ into invisible dark matter candidates, such as those of Refs. [35, 77].

## Summary

The standard model (SM) production of $\text {t} {}{\overline{\text {t}}} \text {t} {}{\overline{\text {t}}}$ has been studied in data from $\sqrt{s} = 13\,\text {TeV}$ proton–proton collisions collected using the CMS detector during the LHC 2016–2018 data-taking period, corresponding to an integrated luminosity of 137$\,\text {fb}^{-1}$. The final state with either two same-sign leptons or at least three leptons is analyzed using two strategies, the first relying on a cut-based categorization in lepton and jet multiplicity and jet flavor, the second taking advantage of a multivariate approach to distinguish the $\text {t} {}{\overline{\text {t}}} \text {t} {}{\overline{\text {t}}}$ signal from its many backgrounds. The more precise multivariate strategy yields an observed (expected) significance of 2.6 (2.7) standard deviations relative to the background-only hypothesis, and a measured value for the $\text {t} {}{\overline{\text {t}}} \text {t} {}{\overline{\text {t}}}$ cross section of $12.6^{+5.8}_{-5.2}\,\text {fb}$. The results based on the two strategies are in agreement with each other and with the SM prediction of $12.0^{+2.2}_{-2.5}\,\text {fb}$ [1].

The results of the boosted decision tree (BDT) analysis are also used to constrain the top quark Yukawa coupling $y_{\text {t}}$ relative to its SM value, based on the $|y_{\text {t}} |$ dependence of $\sigma (\hbox {pp} \rightarrow \text {t} {}{\overline{\text {t}}} \text {t} {}{\overline{\text {t}}}$) calculated at leading order in Ref. [2], resulting in the 95% confidence level ($\text {CL}$) limit of $|y_{\text {t}}/y_{\text {t}}^{\mathrm {SM}} | < 1.7$. The Higgs boson oblique parameter in the effective field theory framework [11] is similarly constrained to $\hat{H} < 0.12$ at 95% $\text {CL}$. Upper limits ranging from 0.1 to 1.2 are also set on the coupling between the top quark and a new scalar ($\phi$) or vector (${\hbox {Z}}^{\prime }$) particle with mass less than twice that of the top quark ($m_\text {t}$) [9]. For new scalar ($\text {H}$) or pseudoscalar ($\text {A}$) particles with $m > 2m_\text {t}$, and decaying to $\text {t} {}{\overline{\text {t}}}$, their production in association with a single top quark or a top quark pair is probed. The resulting cross section upper limit, between 15 and 35$\,\text {fb}$ at 95% $\text {CL}$, is interpreted in the context of Type-II two-Higgs-doublet models [4–6, 75] as a function of $\tan \beta$ and $m_\mathrm {\text {H}/\text {A}}$, and in the context of simplified dark matter models [7, 8], as a function of $m_\mathrm {\text {H}/\text {A}}$ and the mass of the dark matter candidate.

## Data Availability

This manuscript has no associated data or the data will not be deposited. [Authors’ comment: Release and preservation of data used by the CMS Collaboration as the basis for publications is guided by the CMS policy as written in its document "MS data preservation, re-use and open access policy" (https://cmsdocdb.cern.ch/cgi-bin/PublicDocDB/RetrieveFile?docid=6032&filename=CMSDataPolicyV1.2.pdf&version=2).]
